# Dysbiosis in Peripheral Blood Mononuclear Cell Virome Associated With Systemic Lupus Erythematosus

**DOI:** 10.3389/fcimb.2020.00131

**Published:** 2020-04-06

**Authors:** Gangqiang Guo, Lele Ye, Xinyu Shi, Kejing Yan, Jingjing Huang, Kangming Lin, Dong Xing, Sisi Ye, Yuqing Wu, Baoqing Li, Chaosheng Chen, Xiangyang Xue, Huidi Zhang

**Affiliations:** ^1^Department of Microbiology and Immunology, Institute of Molecular Virology and Immunology, Institute of Tropical Medicine, School of Basic Medical Sciences, Wenzhou Medical University, Wenzhou, China; ^2^Department of Gynecologic Oncology, Wenzhou Central Hospital, Wenzhou, China; ^3^Second Clinical College, Wenzhou Medical University, Wenzhou, China; ^4^Department of Laboratory Medicine, Second Affiliated Hospital & Yuying Children's Hospital, Wenzhou Medical University, Wenzhou, China; ^5^Department of Nephrology, First Affiliated Hospital, Wenzhou Medical University, Wenzhou, China

**Keywords:** systemic lupus erythematosus (SLE), virome, metatranscriptomic analysis, peripheral blood mononuclear cells (PBMCs), marker

## Abstract

**Objective:** Pathogen infection plays a role in the development and progression of systemic lupus erythematosus (SLE). Previous studies showed that peripheral blood mononuclear cells (PBMCs) harbor many viral communities. However, little is known about the viral components and the expression profiles of SLE-associated virome. We aimed to identify viral taxonomic markers of SLE that might be used in the detection of disease or in predicting its outcome.

**Methods:** Non-human sequence data from high-throughput transcriptome sequencing of PBMC samples from 10 SLE patients and 10 healthy individuals were used for taxonomic alignment against an integrated virome reference genome database. Based on abundance profiles of SLE-associated virome species, genera, or host, Random Forests model was used to identify the viruses associated with SLE diagnostic markers. Spearman's correlation and functional clustering was used to analyze the interaction of candidate virome dysbiosis and SLE-associated differentially expressed genes.

**Results:** A total of 419 viruses (38 human associated viruses, 350 phage, and 31 other viruses) was detected and the diversity of the PBMC virome was significantly increased in patients with SLE compared to the healthy controls (HCs). Viral taxa discriminated the cases from the controls, with an area under the receiver operating characteristic curve of 0.883, 0.695, and 0.540 for species, genus, and host, respectively. Clinical subgroup analysis showed that candidate PBMC viral markers were associated with stable- and active-stage SLE. Functional analyses showed that virome dysbiosis was mainly relevant to cellular and metabolic processes.

**Conclusion:** We identified virome signatures associated with SLE, which might help develop tools to identify SLE patients or predict the disease stage.

## Introduction

Systemic lupus erythematosus (SLE) is a multi-system autoimmune disease, which is influenced by complex genetic and environmental (microbiota) factors (Hom et al., 2008). The incidence rates of SLE range from ~1 to 10 per 100,000 person-years and prevalence rates vary from ~20 to 70 per 100,000 person-years, worldwide. The incidence rates for women are ~10-times higher than those for men (Pons-Estel et al., 2010). The pathogenesis of SLE remains unclear. Emerging evidence suggests that microbiota plays an important role in the development of several autoimmune diseases, including SLE (Guo et al., 2018b), type 1 diabetes (Wen et al., 2008), rheumatoid arthritis (Wu et al., 2010), and multiple sclerosis (Berer et al., 2011; Lee et al., 2011). Recent studies have shown that the microbiota mainly protects the intestine against colonization by exogenous pathogens and potentially harmful indigenous microorganisms by directly competing for limited nutrients and by modulating the host immune responses (Kamada et al., 2013). Conversely, pathogens have developed strategies to promote their replication in the presence of competing microbiota. Breakdown of the normal microbial community increases the risk of pathogen infection, the overgrowth of harmful pathobionts, and inflammatory diseases (Kamada et al., 2013). Most therapeutic strategies targeting the microbiome, such as fecal microbial transplantation and administration of probiotics and prebiotics, aim at modulating the microbial community (Lim et al., 2015). In this context, we speculate that dysbiosis of microbiota might participate in the onset and progression of SLE.

The human virome is a subset of the human microbiome, which comprises all microscopic organisms that may be found on and inside the human body, both in health as well as in disease conditions (Mitchell et al., 2016). Increasing evidence indicates that the virome, a diverse community of DNA and RNA viruses and bacteriophages of eukaryotes (Lim et al., 2015), plays an important role in human health. Previous research has consistently shown the association of Epstein-Barr virus (EBV) and cytomegalovirus (CMV), which are eukaryotic DNA viruses, with SLE (Kaul et al., 2016; Watad et al., 2017; Guo et al., 2018b). This further indicates that virome may be related to the occurrence and development of SLE.

PBMCs, which can readily migrate via blood throughout the body, are not only crucial for the spread of viruses to almost all tissues and organs following primary infection but are central to the entire strategy for their persistence in the body (Stevenson et al., 2014). Ultrastructural anomalies in PBMCs of patients with SLE indicated their involvement in this disease (Bariety et al., 1971). Although 94 different viruses were identified at DNA level in the blood virome of 8,240 healthy individuals (Moustafa et al., 2017) and the virome associated with childhood leukemia was analyzed in 14 pediatric acute lymphocytic leukemia patients (Bartenhagen et al., 2017), there has been no metatranscriptomic analysis of the PBMC virome in SLE patients, till date.

Previous studies have shown the presence of polyomaviruses, BK and JC, in PBMCs of healthy blood donors using semi-nested polymerase chain reaction (PCR) and qPCR (Haghighi et al., 2019). TTV DNA was detected in PBMCs of healthy adults by real-time PCR (Naganuma et al., 2008). GB virus C (GBV-C/HGV), enterovirus, human herpesvirus-8, and HIV-1 were detected in PBMCs of some chronic hepatitis B, C, and D (Madejon et al., 1997), hemodialysis (Cabrerizo et al., 1999), type 1 diabetes (Yin et al., 2002), Castleman's disease (Yamasaki et al., 2003), and immunodeficiency disease patients (Ou et al., 1988; Saksela et al., 1995; De Milito et al., 1996) by reverse-transcription nested PCR, PCR, and RT-PCR. However, only one or few viruses can be detected simultaneously using these experimental techniques. Because of the diversity of viruses, it is difficult to detect all viral pathogens using routine methods of detection and to make a definitive diagnosis. Currently, high-throughput sequencing technology has become a powerful method for detecting pathogens. It allows for the detection of pathogens without any prior genetic information. RNA-seq data of 239 cases of head and neck squamous cell carcinoma (HNSCC) available in the Cancer Genome Atlas database was used to detect the known viruses (26). In another study, whole-genome sequencing of blood DNA from 8,240 individuals was done to analyze the virome (15). These studies provide essential insights and clearly demonstrate the potential of high-throughput sequencing technologies (27) and motivated us to perform an unbiased survey of viral expression in PBMCs collected from SLE patients.

We performed a metatranscriptomic association study of the virome in PBMCs of SLE patients, with particular attention to the alterations in the PBMC virome. To the best of our knowledge, this is the first report showing the taxonomic composition of the PBMC virome in SLE patients. We also show the interactions between the candidate viral markers and differentially expressed human genes (DEGs), and the association of these DEGs with cellular and metabolic processes. Our results support the characteristics of the PBMC virome dysbiosis in SLE patients, and provide potential markers for the diagnosis of SLE.

## Materials and Methods

### Study Population

Fifteen SLE patients admitted to the Department of Rheumatology and Nephrology, First Affiliated Hospital of Wenzhou Medical University, China, between February 2017 and December 2019, were enrolled in this study. All the patients fulfilled the American College of Rheumatology 1997 criteria for SLE (Hochberg, 1997). The disease activity was assessed according to the Systemic Lupus Erythematosus Disease Activity Index (SLEDAI) (Bombardier et al., 1992) using blood samples obtained before the patients were administered glucocorticoids and immunosuppressive agents. Although patients were included at time of diagnosis, they were not all naïve at time of blood collection. Patients with SLEDAI ≥ 5 were defined to have “active disease,” and those with SLEDAI < 5 were defined to have “stable disease.” Fifteen age- and sex-matched healthy controls (HCs), without arthralgia, heart failure, renal failure, or autoimmune disease, and free from other inflammatory conditions, were recruited based on records available from the Second Affiliated Hospital & Yuying Children's Hospital of Wenzhou Medical University. The research protocol was approved by the Medical Ethical Committees of the First and Second Affiliated Hospitals of Wenzhou Medical University. All subjects who participated in this research provided written informed consent.

### Isolation of PBMCs and RNA Extraction

PBMCs were isolated from SLE patients and HCs using human peripheral blood lymphocyte separation medium (Tianjin Hao Yang Biological Manufacture, Tianjin, China) within 4 h of the collection of blood samples. Total RNA was extracted from each sample using TRIzol reagent (Invitrogen Life Technologies®, Grand Island, NY, USA). The isolated RNAs were digested with Dnase I (Invitrogen™, Waltham, MA, USA) to remove the residual DNA, and were suspended in 25 μL of DNase/RNase-free water. Total RNA from each sample was quantified and its quality was assessed by Agilent 2100 Bioanalyzer (Agilent Technologies, Palo Alto, CA, USA), NanoDrop (Thermo Fisher Scientific Inc., Waltham, MA, USA), and 1% agarose gel electrophoresis. Total RNA (1 μg) with an RNA integrity number above 7 was used for library preparation.

### Library Preparation and Paired-End Sequencing

RNA libraries for next generation sequencing (NGS) were constructed with ten SLE patients and ten HCs according to the manufacturer's protocol (NEBNext® Ultra™ Directional RNA Library Prep Kit for Illumina®, San Diego, CA, USA). The rRNA was removed from the total RNA samples using Ribo-Zero™ rRNA removal Kit (Human/Mouse/Rat) (Illumina) and the remaining RNA was then fragmented and reverse-transcribed. The first strand cDNA was synthesized using ProtoScript II Reverse Transcriptase with random primers and actinomycin D. The second-strand cDNA was synthesized using Second Strand Synthesis Enzyme Mix (including dACG-TP/dUTP). The double-stranded cDNA was purified by AxyPrep Mag PCR Clean-up (Axygen) and then treated with End Prep Enzyme Mix to repair both the ends. dA-tailing was performed in one reaction, which was followed by T-A ligation to add adaptors to both the ends. Size selection of adaptor-ligated DNA was then performed using AxyPrep Mag PCR Clean-up (Axygen), and ~360 bp fragments (with an approximate insert size of 300 bp) were recovered. Libraries with different indices were multiplexed and loaded on an Illumina HiSeq instrument according to the manufacturer's instructions (Illumina). Sequencing was carried out using a 2 × 150 paired-end (PE) configuration; image analysis and base calling were conducted by the HiSeq Control Software (HCS) + OLB + GAPipeline-1.6 (Illumina) on the HiSeq instrument.

### Workflow for the Identification of Viral Sequences

Viral sequences in SLE RNA-seq data were detected as previously described, with some modifications (Bartenhagen et al., 2017; Moustafa et al., 2017; Nakatsu et al., 2018). In brief, read pairs with adapter sequences, N-containing bases at the 5′- or 3′-end, and low quality data and contaminating sequences and average sequence quality (Phred score) below 30 were removed using Cutadapt (version 1.9.1) keeping the following parameters, –e 0.1 –q 20,20 e 0.1 –q 20,20 –m 10 –max–n = 0.1 m 10 –max–n = 0.1. The remaining reads were subsequently aligned using Burrows–Wheeler Aligner (BWA, version 0.7.15) in paired-end mode with default parameter settings to map all the patient datasets against human reference genome build hg19. Unmapped reads were collected using samtools (samtools view –f4) and further filtering of human content using Bowtie2 (version 2.2.6). The scoring and penalty parameters were set as follows: bowtie2 —very-sensitive —mp 1,1 —rdg 2,1 —rfg 2,1 —score–min L,0, −0.15. All the reads, which were unmapped after the BWA and Bowtie2 alignment, were aligned against a more comprehensive database comprised of NCBI RefSeq viral reference genomes (9,334 viruses and phages) using blastn (2.3.0), with an evalue <10^−5^ (Supplementary Figure 1). The version of the NCBI RefSeq viral reference genomes was V82 and was downloaded from ftp://ftp.ncbi.nlm.nih.gov/refseq/release/viral/viral.2.1. genomic.fna.gz and ftp://ftp.ncbi.nlm.nih.gov/refseq/release/viral/viral.1.1. genomic.fna.gz. To quantify the load of viruses in the samples the blast hit results were collected and the best hits selected for each read using a custom script (Bioperl). Thereafter, randomly selected viral reads of the human associated viruses were manually and visually verified by searching (blastn) against the NCBI nucleotide collection (nr/nt) database (online) and by aligning the reads to the corresponding viral genomes. Moreover, all reads, which were unmapped after the BWA and Bowtie2 alignment, were aligned to the reference genomes of seven human associated viruses with Bowtie2 (bowtie2 –very-sensitive) instead of blastn (Bartenhagen et al., 2017). The reference genomes of seven human associated viruses were selected as follows: human herpesvirus 2 strain HG52 (NC_001798.2), human herpesvirus 4 (NC_009334.1), human herpesvirus 4 complete wild type genome (NC_007605.1), human herpesvirus 5 strain Merlin (NC_006273.2), human herpesvirus 6A (NC_001664.4), human herpesvirus 6B (NC_000898.1), human herpesvirus 7 (NC_001716.2), and human herpesvirus 8 (NC_009333.1). This method also could only detect the human herpesvirus 4 type 2, human herpesvirus 4 (strain B95-8), human herpesvirus 5 strain Merlin, and human herpesvirus 8, as was the case when blast alignment tool was used, indicating that blastn is indeed a useful tool for identification of viral sequences (Altschul et al., 1990; Pruitt et al., 2007; Moustafa et al., 2017).

### Calculation of Viral Abundance

Viral abundance was determined as described previously (Moustafa et al., 2017). The normalized abundance (viral genomes per human diploid genome) of a virus in an SLE sample was estimated with the following equation:

$$\begin{array}{l} virus\;abundance=\frac{2\times \frac{\left(number\;of\;reads\;mapped\;to\;viral\;genome\right)}{\left(virus\;genome\;size\right)}}{\frac{\left(number\;of\;reads\;mapped\;to\;human\;genome\right)}{\left(human\;genome\;size\right)}} \end{array}$$

### Ordination Analysis

We performed correspondence analysis (CA) and distance-based redundancy analysis (dbRDA) and assessed the significance of separation among groups by permutational multivariate analyses of variance (PERMANOVA) using the vegan package in R. Ordination analyses were conducted and visualized using the ggord, ggpubr, and ggplot2 packages in R, respectively.

### Microbial Feature Selection and Construction of Machine Learning Models

To identify a robust set of PBMC virome features for disease classification of discovery metatranscriptomic samples, we designed a parallel Random Forests model-based backward feature elimination (RF-BFE) algorithm guided by ranked feature importance scores (mean decrease in accuracy, MDA), with built-in hyper-parameter tuning through random search using the R implementation of Breiman and Cutler's Random Forests algorithm (Breiman, 2001). Heatmap was used to visualize the data using the heatmap2 package in R. In each complete round of backward feature elimination with a random permutation of mtry, ntree, and nodesize parameters, we computed stepwise average area under the curve (AUC) with balanced class weights and feature importance scores over 10-times repeated 10-fold cross-validations of RF-BFE model (RF-BFE-AUC). Significance of area under the receiver operating characteristics curve (AUROC) under the null hypothesis of random predictive value was evaluated using ROCR package in R. Dirichlet multinomial modeling and Bayesian ordination of virome samples was performed as previously described (Holmes et al., 2012; Ren et al., 2017).

### Spearman's Correlation Between Genes and Candidate Viral Markers and GO and KEGG Pathway Analysis

To infer the regulatory network of genes and virus communities, we performed correlations on significant DEGs and candidate viral markers. For determining the correlations, we implemented the Hmisc package in R within the Spearman's correlation, and evaluated the significance of differential correlations. The Spearman's correlation between genes and viruses was visualized using the heatmap.

The DEGs in the taxonomic pairs with significant differential correlations between two groups, as determined by Spearman's statistics (*P* < 0.05) and correlation coefficient (≥0.6), were selected for PANTHER analysis (http://www.pantherdb.org/). The gene ontologies (GO) of molecular functions, and biological processes of correlated genes were analyzed. The lower the *P-*value, the more significant was the GO term (*P* < 0.05 was considered statistically significant). Biological networks were determined using Analyze Networks (AN) algorithm with default settings and analyzed using MetaCore (https://portal.genego.com/).

## Results

### Expansion of the PBMC Virome in SLE Patients

**Figure 1:**
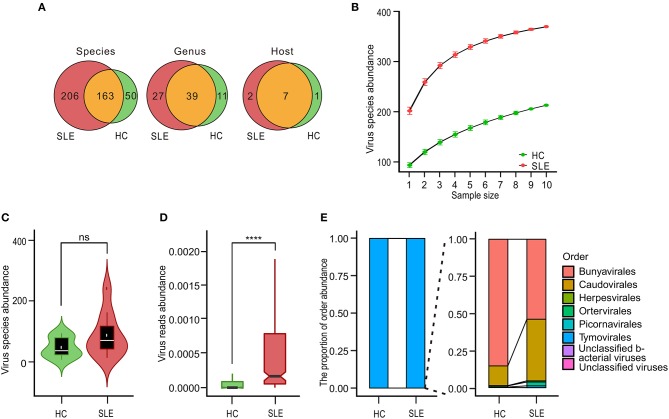
Variations in systemic lupus erythematosus (SLE)-associated peripheral blood mononuclear cell (PBMC) virome community. **(A)** Venn diagrams of virome species, genera, and host diversity between SLE patients and healthy controls (HCs). **(B)** Rarefaction plot showing the association of virus species diversity and sequencing depths for viral species in PBMCs of SLE and HC subjects. **(C)** Comparison of species diversity of virus communities in the disease state. White dots and black horizontal lines on integrated boxplots represent the mean and median values, respectively. ns, No significant difference. **(D)** Comparison of the abundance of virus communities in the disease state. Black horizontal lines on integrated boxplots represent the median values. ****P < 0.0001. **(E)** Relative sample composition and breakdown of viral metatranscriptomic sequences classified at order-level when compared with viral counterparts. Bar charts depicting taxonomic landscape of viral orders and overall relative proportions compared with virus taxa between HC and SLE samples.

We sequenced the transcriptome of PBMCs from 10 SLE patients and 10 HCs. On an average, each sequencing sample generated 1 billion reads. The majority (94%) of the reads were successfully mapped to the hg19 human reference genome. Among the remaining reads, similarity search assigned <0.01% reads to viruses for most of the samples. According to the virome taxonomic information in the metatranscriptomic analysis, we first explored the characteristics of the viromes from the aspects of species, genus, and host (Supplementary Tables 1, 2); the process for analysis is detailed in Supplementary Figure 1. A total of 419 species (38 human associated viruses, 350 phage, and 31 other viruses) of viruses were detected in SLE and HC PBMCs. These viruses belonged to 77 genera from 10 hosts. Among them, there were 369 and 213 species, 66 and 50 genera, and 9 and 8 hosts for viruses in the SLE and HC samples, respectively. The community composition of 163 species, 39 genera, and 7 hosts were the same in SLE and HC PBMCs. Moreover, the number of viruses in PBMCs of SLE patients was more than in those of HCs, with respect to species, genera, and hosts (Figure 1A). Rarefaction plot further showed that the number of virus species detected was increased as the sampling increased (Figure 1B). However, we did not find any significant increase in the diversity of PBMC viromes (Figure 1C). In spite of this, interestingly, we observed a significant increase in the abundance of viral reads in PBMCs (*P* = 7.03 ×1 0^−4^, Figure 1D). Moreover, we divided 419 detected viruses into eight orders according to the International Committee on Taxonomy of Viruses database. The proportion of the abundance of orders between the PBMCs of SLE patients and HCs was also analyzed. The results showed that the Tymovirales order was dominant in the two groups with the largest proportion (Figure 1E, left-panel). Compared to the PBMCs of HCs, the proportions of Caudovirales, Herpesvirales, Ortervirales, Picornavirales, and unclassified bacterial viruses, but not of Bunyavirales and unclassified viruses, were increased in the PBMCs of SLE patients (Figure 1E, right-panel).

### PBMC Virome Can Be Used to Distinguish SLE Patients

**Figure 2:**
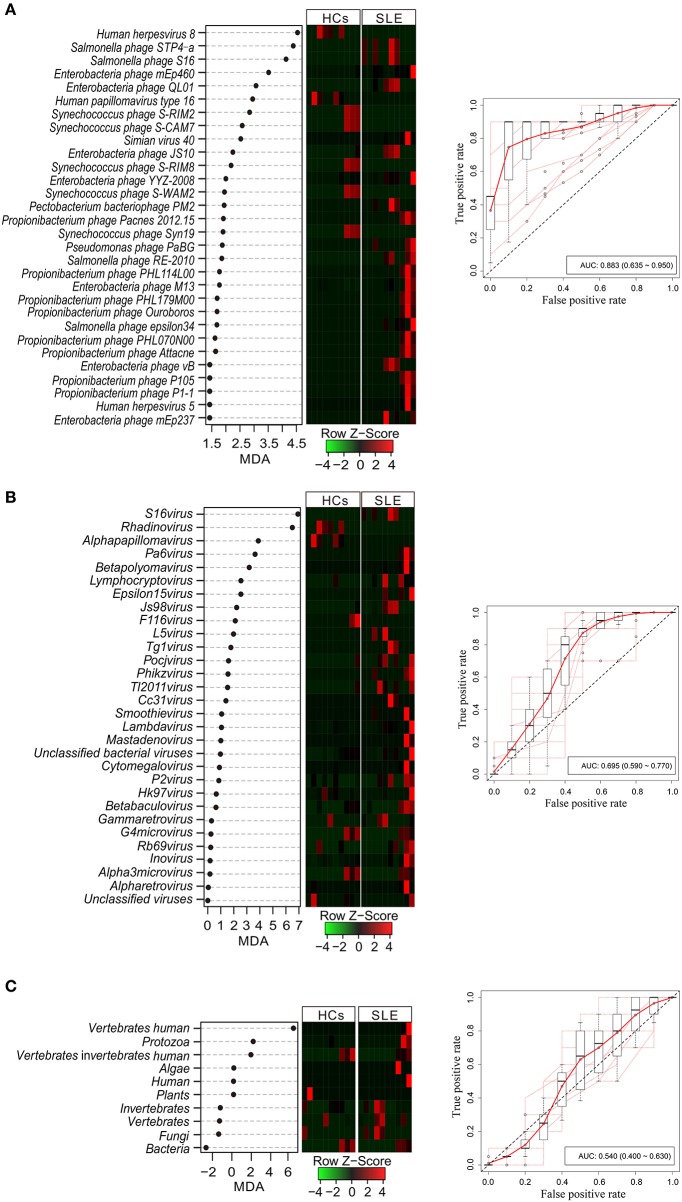
Performance of virome markers in the diagnosis of systemic lupus erythematosus (SLE) at species **(A)**, genus **(B)**, and host **(C)** levels. Dot-plot showing the average importance scores from 1000 iterations of discovery model fit and ranking of most discriminatory virome species, genera, and host-level markers identified by Random Forests-based backward feature selection (left panel). Heatmap shows the abundance of virome markers (middle panel). Internal 10-times repeated 10-fold cross-validations of SLE virome-based metatranscriptomic classifier. Red line represents the average true and false positive rates. Out dots depict each individual round of tenfold model cross-validations summarized by boxplots (right panel).

To determine the potential of viral taxa in discriminating the disease status, we identified a minimal set of 30 viral species (4 human associated viruses and 26 phage viruses) and 30 genus and 10 (all) hosts that maximally differentiated SLE patients from HCs by Random Forests-based backward feature selection (Figures 2A–C, left-panels, respectively). Random Forests (RF) models trained with the optimal set of markers generated a percent out-of-bag (OOB) error rate of 41.14 ± 0.07, 45.14 ± 0.03, and 46.00 ± 0.04 with species, genus, and host, respectively (means ± s.d.; *n* = 1000). Among the top-ranked viral species, human herpesvirus 8 had a mean fold importance increase of 1.02 compared to *Salmonella* phage STP4a. Other discriminatory markers included *Salmonella* phage S16, Enterobacteria phage mEp460, and Enterobacteria phage QL01, which were the top-five-ranked viral species. Among the genera, S16 virus was the top-ranked with an MDA of 6.82. Other discriminatory markers included Rhadinovirus, Alphapapillomavirus, Pa6 virus, and Betapolyomavirus, which had MDAs ranging from 3.07 to 6.38 (mean; *n* = 1000). However, only three MDA values (6.42, 2.04, and 1.78) of viral hosts were >0. The analysis of abundance demonstrated disease-specific enrichment and depletion of these viral markers in SLE with species, genus, and host (Figures 2A–C, middle-panels). To assess the accuracy of model classification, we subjected average incidences from 10-times repeated 10-fold cross-validations to ROC analysis, which generated an AUROC of 0.883, 0.695, and 0.540 with species, genus, and host, respectively (Figures 2A–C, right panels). We further analyzed the performance of the cohort classifiers with the low and high abundance of species, genus, and host between the two groups, respectively (AUROC_species−high_ = 0.844, AUROC_species−low_ = 0.789, AUROC_genus−high_ = 0.623, AUROC_genus−low_ = 0.688, AUROC_host−high_ = 0.536, AUROC_host−low_ = 0.388, Supplementary Figure 2), which indicated that species had an obvious classification ability.

### Clinical Stage–Dependent Development of the PBMC Virome in SLE

**Figure 3:**
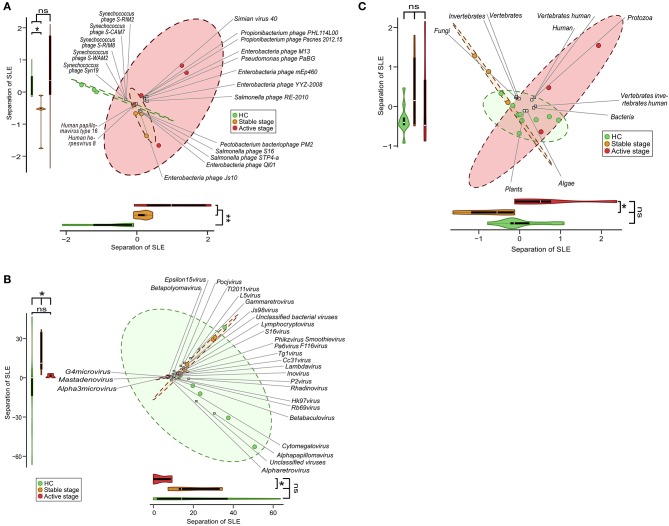
Clinical stage-associated dysbiosis of the peripheral blood mononuclear cell (PBMC) virome in systemic lupus erythematosus (SLE). Biplot summarizing the redundancy analysis (dbRDA) of virome profiles at species- **(A)**, genus- **(B)**, and host-levels **(C)**. Projection axes were assessed individually by Wilcoxon's rank-sum tests. ns, no significant difference; *P < 0.05; **P < 0.01.

Given the discriminatory potential of the PBMC virome markers, we tested whether there were any disease specific changes in the PBMC virome. Using high-resolution taxonomic profiles of virome species, genus, and host, we performed CA and dbRDA of the HCs and stage-stratified SLE samples, which included stable-stage SLE samples (*n* = 5) and active-stage SLE samples (*n* = 5). PERMANOVA showed a significant separation with species between HCs and stable SLE stage, and HCs and active SLE stage (Figure 3A). A significant separation between HCs and stable SLE stage, and stable SLE stage and active SLE stage also observed with genus (Figure 3B). However, only a significant separation between stable SLE stage and active SLE stage was found with host (Figure 3C). This was consistent with the finding that viral species and genus can discriminate between HCs and SLE (*P* < 0.05) (Supplementary Figures 3A,B). Unexpectedly, no separation was observed between HCs and SLE with host (Supplementary Figure 3C).

### Association of the PBMC Virome With SLE

**Figure 4:**
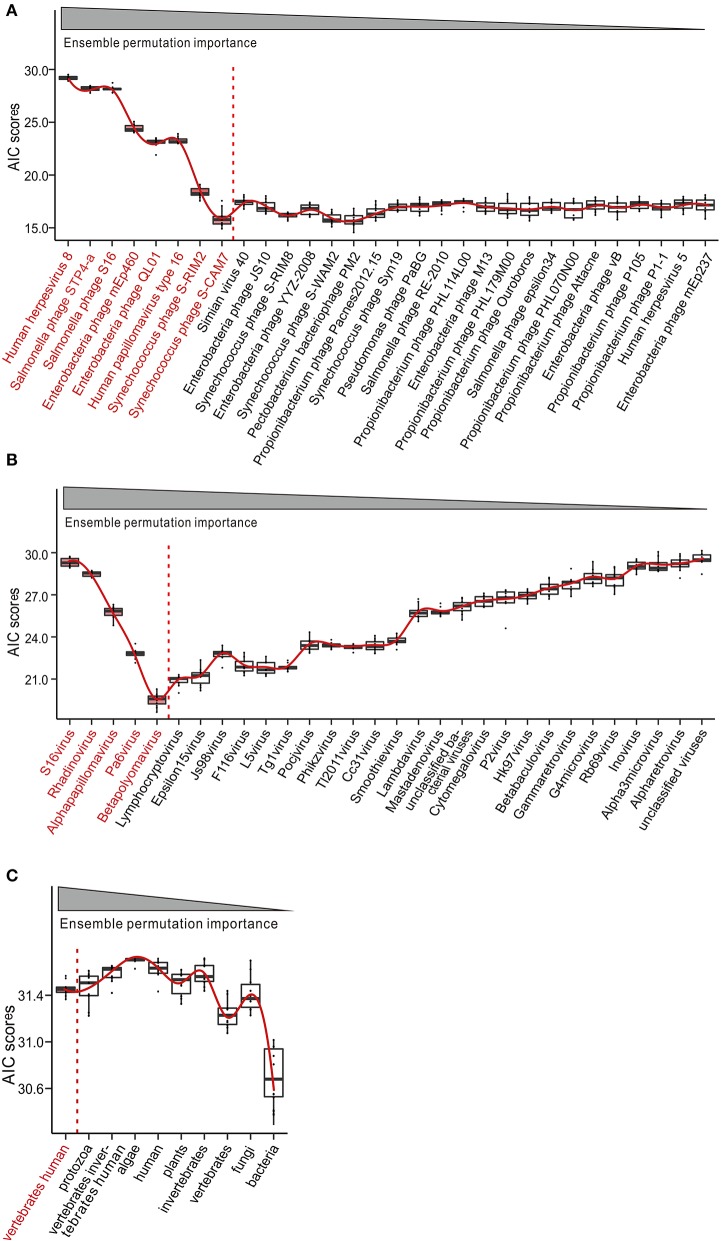
Potential of peripheral blood mononuclear cell (PBMC) virome taxa for the diagnosis of systemic lupus erythematosus (SLE). Construction and evaluation of logistic regression (LR) classifiers with ensemble permutation importance measure-guided forward selections of diagnosis predictors at species **(A)**, genus **(B)**, and host **(C)** levels. Akaike Information Criterions (AIC) were computed using out-of-bag (OOB) predicted morbidity scores from 1,000 iterations of LR modeling. Red line indicates the curve fitting. Color-coded taxa (black) represent the optimal diagnostic predictors.

Having observed the disease-specific alterations, we sought to know whether the SLE-associated viral markers of species, genus, and host would be effective for disease diagnosis. To address this question, we built logistic regression (LR) models through stepwise inclusion of viral markers of species, genus, and host in the decreasing order of importance. In this OOB forward selection, we obtained a combination of top-eight, five, and one markers of species, genus, and host, respectively, which optimized the performance of the LR model (Figure 4). We also evaluated the SLE-associated viral markers of species, genus, and host in the group of high or low abundance of viruses in SLE patients, respectively. The results showed that the markers were useful for diagnosis (Supplementary Figure 4). The results showed that seven and four viruses, which also contained the top-eight viruses (Figure 4A, species), could effectively diagnose among the high and low abundance of viruses, separately. Besides, we could see that the abhorrent viruses (Simian virus 40, Enterobacteria phage JS10, and Enterobacteria phage YYZ 2008) seldom affected the LR model (Supplementary Figure 4A, left panel). In addition, we found a similar result in the genus analysis (Supplementary Figure 4B). Unexpectedly, we did not find any obvious correlation in the host analysis (Supplementary Figure 4C).

### Interactions of Candidate PBMC Viral Markers and DEGs in SLE

**Figure 5:**
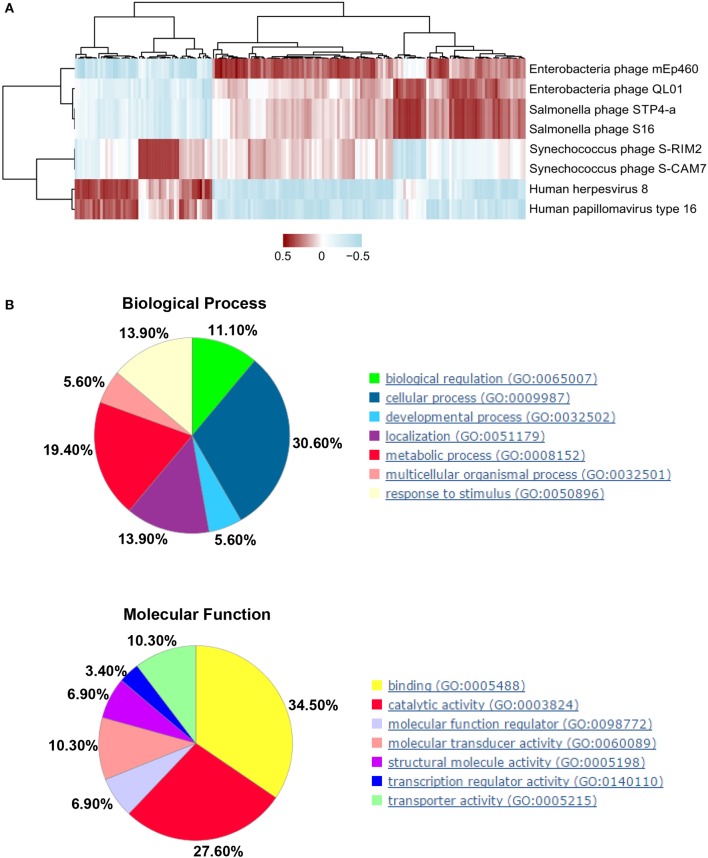
Interactions between significant differentially expressed genes (DEGs) in human host and candidate peripheral blood mononuclear cell (PBMC) virus markers in systemic lupus erythematosus (SLE). **(A)** Correlations between significant human DEGs and candidate viral markers between health control (HCs) and SLE subjects, as determined by Spearman's statistics (*P* < 0.05) and correlation coefficient (≥ |0.6|), were selected for the visualizations. Clustered heatmap used the complete method with correlation showing the strength of correlations. **(B)** Gene Ontology (GO) analysis: biological processes (upper panel) and molecular function (lower panel).

Next, we explored the association between DEGs and candidate viral markers in SLE. We built disease-specific inference networks of direct taxonomic correlations, controlling for abundance data composition (see Methods). Top-eight viruses from the reliable models constructed, as described in the previous section (Figure 4), and 585 DEGs were selected, as described in Methods for correlation analysis. After correlation tests, we chose the correlation coefficients greater than |–/+0.6| and with a *P* < 0.05 for visualization (Figure 5A, Supplementary Table 3). We observed that there were 177 DEGs that significant correlated with the top-eight viruses in the abovementioned analysis. Furthermore, functional analysis was performed for these DEGs, and biological process analyses showed that the 177 DEGs were closely related to cellular (GO: 0009987) (30.6%) and metabolic (GO:0008152) (19.4%) processes. Molecular function analysis showed that the DEGs were closely related to binding (GO: 0005488) (34.50%) and catalytic activity (GO: 0003824) (27.60%) (Figure 5B, Supplementary Table 4). Furthermore, GO process analysis indicated that the virome mainly participated in cytoskeleton organization, signal transduction, and metabolism of phosphate-containing compounds (Supplementary Figure 5A). The AN algorithm, used with default settings, showed that the most relevant biological networks of the DEGs were regulation of signal transduction (93.9%), cell surface receptor signaling pathway (87.8%), regulation of cell proliferation (85.7%), gland development (61.2%), and canonical Wnt signaling pathway (42.9%) (Supplementary Table 5, Supplementary Figure 5B).

## Discussion

In SLE, disease initiation and development are influenced by a myriad of genetic and environmental factors. Viral infections, which impinge on the human immune system, may undoubtedly affect the etiology and/or pathogenesis of a given autoimmune disease in certain individuals (Nelson et al., 2014). Analysis of a multi-order of PBMC virome creates new research opportunities for understanding the SLE pathogenesis. Previous research showed that many viruses are present in PBMCs. Human foamy virus (HFV) (Sun et al., 2006), Epstein-Barr virus (EBV) (Han et al., 2018), human T lymphotropic virus type I (HTLV) (Akimoto et al., 2007), human cytomegalovirus (HCMV) (Chen et al., 2015), and human herpesvirus 6 (HHV6) (Broccolo et al., 2013) exist in PBMCs or peripheral blood leukocytes of SLE patients. Although some viruses have been detected in PBMCs of SLE, virome diversity and variations in the PBMC microbial flora remain unexplored.

Although NGS is used for the discovery of a multitude of viruses, owing to the nature of the sequencing method, there is limited amplification of the viral genome because of significant competition from the larger human genome. On the other hand, the virome study is based on viral genome analysis, which is not conducive for detecting viruses because of the small size and low copy number of viral genome. Generally, viruses can inhibit host cell transcription and promote viral gene expression following infection. For this reason, compared to the metagenome analysis, virome metatranscriptome analysis can significantly improve the sensitivity of virome detection.

Here, we showed the alterations in the PBMC virome using NGS, indicating the multifaceted role of the microbiome in the development of SLE. We identified 419 different viruses, including 38 associated human viruses. Compared to the results of previous studies, unexpectedly, we did not detect HFV, HHV6, and HTLV in PBMCs of SLE patients. We also did not detect HHV8 (Naganuma et al., 2008; Broccolo et al., 2013), but EBV (Han et al., 2018) and HHV5 (Chen et al., 2015) were detected in PBMCs of SLE patients, as in previous studies. In addition, the diagnostic capabilities of two viruses, namely HHV5 and HHV8 (screened by Random Forests-based backward feature selection; Figure 2A), which might serve as SLE markers, were selected and discussed in this study based on previously published data. In our previous study, using two RNA-Seq techniques in combination with RT-PCR, HHV5 infection was identified in the PBMCs of SLE patients, and the expression level was significantly higher in 73 SLE patients than in 75 HCs (*P* < 0.001) (Guo et al., 2018b). Serum samples from SLE patients (*n* = 108) and matched controls (*n* = 122) were collected, and the prevalence of HHV8 was compared using a virus-specific nested PCR. There was significant difference in the prevalence of HHV8 DNA between SLE patients and HCs (11 of 107 vs. 1 of 122, *P* = 0.001) (Sun et al., 2011). However, Balada et al. found that only 2 out of the 82 samples analyzed yielded the specific HHV8 PCR product in nested PCR of DNA obtained from SLE whole blood samples (Balada et al., 2015). Tung et al. found that no HHV8 DNA was detected by real-time PCR in the PBMCs of SLE patients (Tung et al., 2013). These differences in the results might have been caused by the differences in SLE samples and the molecules (DNA or RNA) that were detected, heterogeneity of SLE, different genes of the virus, and the number of samples used.

In this study, the abundance of HHV5 in the PBMCs of SLE patients was also higher than in the PBMCs of HCs. Besides, HHV8 was more frequently present in HCs, as reported previously. This preliminary evidence shows that the expression levels of HHV5 and HHV8 (Supplementary Table 2) are in good agreement with previous reports and can be used as SLE markers. Moreover, the HHV4 burden in PBMCs was over 15-fold greater in SLE patients than in HCs individuals, as determined using the PCR assay, suggesting that HHV4 infection is abnormally regulated in SLE (Moon et al., 2004). In this study, we also found that HHV4 is more frequently present in SLE patients and is associated with SLE (Supplementary Table 2). Therefore, we believe that our methods and results are reliable and reproducible. Nevertheless, different genes of the virus are differentially expressed (gene expression status or expression levels) in different disease conditions. Therefore, it would be inadequate to select a single viral gene and verify each by quantitative PCR to determine whether the virus can be used as a universal diagnostic marker. In this study, we evaluate the virus's overall abundance as a diagnostic marker of systemic lupus erythematosus. Therefore, these results require further validation using more viral species, samples, and methods.

In addition to a wide representation of human herpesviruses and anelloviruses in these 38 human associated viruses, we identified three different viruses, including Shamonda virus, simian virus 40, and human papillomavirus type 16. Shamonda virus belongs to the Simbu group of the genus *Orthobunyavirus* and family Bunyaviridae; several viruses of this group have been reported to cause diseases in humans (Yanase et al., 2005). The polyomavirus simian virus 40 is a potent DNA tumor virus, and mounting evidence suggests that it is an emergent human pathogen, which could lead to cancer in humans under natural conditions (Vilchez and Butel, 2004). Shamonda virus and simian virus 40 are predominant in PBMCs of SLE patients; however, human papillomavirus type 16 was only detected in PBMCs of HCs, as was reported previously (Moustafa et al., 2017). However, whether these viruses are implicated in SLE development needs further research.

Furthermore, among the sequences that were mapped to 419 viruses, we identified sequences for 350 bacterial viruses in 83.5% of the participants, as was previously reported for blood/cells (De Vlaminck et al., 2013; Kowarsky et al., 2017; Nguyen et al., 2017). Kowarsky et al. analyzed massive non-human shotgun sequence data from 1351 blood samples collected from 188 patients and assembled 7,190 contiguous regions, of which 3,761 were novel, with little or no sequence homology in any of the existing databases. They identified hundreds of new bacteria and viruses, which represent previously unidentified members of the human microbiome. In particular, a total of 276 (9%) regions from the 2,917 placed novel contigs corresponded to novel viral sequences, which were predominantly either from phages or torque teno viruses (Kowarsky et al., 2017). Moreover, the identification of pathogen-derived sequences from 656 plasma samples, collected from transplant recipients, were also obtained using BLAST against reference database of viral (*n* = 1,401), bacterial (*n* = 1,980), and fungal (*n* = 32) genomes. A total of 0.12% of the uniquely sequenced reads aligned to at least one of the target genomes. It was found that viruses (73%) were more abundantly represented than bacteria (25%) and fungi (2%) in the plasma (De Vlaminck et al., 2013). Moustafa et al. explored the non-human sequence data from whole-genome sequencing of blood from 8,240 individuals and identified 94 different viruses in the blood; they suggested that the remaining 75 viruses mostly reflected extensive contamination of commercial reagents and contamination from the environment (Moustafa et al., 2017). Nguyen et al. also suggested that 31 billion bacteriophage particles are transcytosed across the epithelial cell layers of the gut into the average human body each day (Nguyen et al., 2017). Moreover, some researchers suggested that viruses, bacteria, and fungi release their DNA and RNA into the blood, a phenomenon that has been detected to verify the presence (De Vlaminck et al., 2015) and to measure the alterations in the virome because of the alterations in the state of the immune system (De Vlaminck et al., 2013). In conclusion, these evidences show that a number of microorganisms exist in our blood/cells. Moreover, the presence of three phages was checked in PBMCs from five mixed SLE patients and five mixed normal samples using PCR in this study (Supplementary Figure 5C). The results confirmed that the presence of phage sequences in the PBMCs of SLE patients and HCs was not because of contamination. However, these results suggesting the existence of bacteriophages in PBMCs are only preliminary and need to be further validated using more samples and detection methods.

It has also been previously reported that phages utilizes various mechanisms (such as leaky gut, Trojan Horse, phage display, free uptake) to access the body from the gut; they are disseminated throughout the body and support the establishment of the intrabody phageome (Barr, 2017; Nguyen et al., 2017), acting as a new innate immune system of our body, which might alert the immune system to the presence of potential pathogens (Barr, 2017; Nguyen et al., 2017). Thus, bacteriophages might affect the behavior of PBMCs in SLE patients through these mechanisms, thereby, affecting the pathogenesis of SLE. However, whether alterations in the PBMC virome reflect the changes in the health of the human host, and the mechanism of action involved, remains unclear. Moreover, viral diversity and abundance showed a relative increase in SLE-associated PBMC virome compared to the virome of HCs, indicating that virus infection may constitute an important trigger for SLE and might further aggravate disease progression. In turn, SLE aggravation also promotes virus infection (Guo et al., 2018b). Moreover, it has been suggested in a previous report that the total viral load in the plasma increases with immunosuppression in recipients of organ transplants (De Vlaminck et al., 2013). These evidences lead us to surmise that the presence of more eukaryotic viruses was likely to be the consequence of viral reactivation and higher susceptibility to viral infection due to the immunocompromise of SLE patients (De Vlaminck et al., 2013). Therefore, we think that most viruses found in the SLE patients were probably only bystanders and the result of immunosuppression, and are, therefore, unlikely to be related to SLE pathogenesis. Only few viruses participated in SLE pathogenesis and we only screened the SLE associated viruses, which could be diagnostic markers.

To determine the potential of viral taxa in discriminating the disease status, we identified a minimal set of 30 viral species, 30 genera, and 10 (all) hosts that maximally differentiated SLE patients from HCs by Random Forests-based backward feature selection. ROC analysis, performed to assess the accuracy of the model, showed that the 30 viral species and 30 genera were able to distinguish SLE patients from HCs. Besides considering the influence of disease complications and drug intervention on the composition of the PBMC virome, we unraveled clinical stage–specific taxonomic profiles across the virome that might be useful for modeling the disease progression (Nakatsu et al., 2018). The result also showed the virome species and genera that contribute to the performance of metatranscriptomic SLE disease classifiers. LR classifier was used to further evaluate the diagnostic ability of candidate viral markers of the 30 viral species and 30 genera. The results showed that the top-five-ranked viral species were human herpesvirus 8, *Salmonella* phage STP4a, *Salmonella* phage S16, Enterobacteria phage mEp460, and Enterobacteria phage QL01. Only human herpesvirus 8 was detected in PBMCs from HCs, and the remaining viruses, which are known to infect Gram negative bacteria, including *Salmonella and Escherichia coli*, had higher abundance of reads in PBMCs from SLE patients than in HCs. Bloodstream infections are common in SLE patients and *Salmonella* infection may result in bacteremia or complications in soft tissues with high mortality and *Salmonella* behaves more aggressively in SLE patients (Gallo et al., 2015). Curli-Containing Enteric Biofilms (components of *Escherichia coli* and *Salmonella* species) lead to the activation of proinflammatory innate immune receptors and potentiate SLE (Tursi and Tukel, 2018). Moreover, the top-five-ranked viral genera were S16virus, Rhadinovirus, Alphapillomavirus, Pa6virus, and Betapolyomavirus. Predominant enrichment of the members of S16virus, Pa6virus, and Betapolyomavirus in the PBMCs of SLE patients might represent a new paradigm of trans-kingdom interaction mediated by the disease virome.

As reported previously (Nakatsu et al., 2018), we also observed individual-specific enrichment of members of herpesviridae, which might be related to the infection history of SLE. The susceptibility to HHV5 and HHV4 is more in SLE; these viruses may play an important role in the pathogenesis of SLE in genetically predisposed individuals (Pasoto et al., 2014). We also observed an increase in the abundance of anelloviruses in PBMCs of SLE patients as a result of lowered immunity; this might serve as a biomarker for functional immunocompetence because changes in anellovirus load in the sera have been associated with immunosuppression levels in transplant recipients (Lim et al., 2015). We envision that simultaneous examination of the abundance of herpesvirus and anelloviruses in PBMC samples may help in improving the accuracy of SLE diagnosis given the cost-effectiveness of virome-based SLE detection for population-level screening. However, whether members of herpesviridae play a role in the onset and progression of SLE needs to be ascertained in future research.

In the context of trans-kingdom interactions, we show how the species of candidate viral markers might interact with significant DEGs in SLE patients. The top-eight viruses, which can effectively distinguish SLE patients from HCs by LR analysis (Figure 4 and Supplementary Figure 4), and 585 significant DEGs were selected for correlation analysis. A total of 177 DEGs with correlation coefficients >0.6 and *P* value < 0.05 with the top-eight viruses were selected. Functional analyses further identified cellular process, metabolic process, regulation of signal transduction, and cell surface receptor signaling pathway to be important in the development of autoimmune diseases. In a previous research, we showed that mRNAs differentially expressed between SLE patients and HCs in PBMCs were also enriched in cellular process, metabolic process, and Wnt signaling pathway (Guo et al., 2018a). Immunometabolism in SLE involves dysregulation in many immune cells, including CD4+ T cells, dendritic cells, macrophages, and neutrophils (Morel, 2017). Specific metabolic processes are key checkpoints of effector functions in the immune system, with common as well as cell-specific programs (Morel, 2017). Moreover, 5-hydroxymethylcytosine is also increased in CD4+ T cells in SLE patients in genes involved in critical pathways of WNT signaling (Zhao et al., 2016). The expression of matrix metalloproteinases (MMPs) was reported to be induced by the changes in Wnt signaling, which contribute to the remodeling of extracellular matrix and to the loss of membrane integrity that occurs in lupus nephritis (Wu et al., 2007; Tveita et al., 2008). Moreover, Wnt signaling plays an important role in homeostatic liver metabolism and can crosstalk with metabolic pathways in normal cells, such as differentiating osteoblasts (Liu et al., 2011; Esen et al., 2013). These results indicate that regulation of metabolic pathways may represent a general mechanism contributing to the wide-ranging functions of Wnt signaling, which may be involved in the occurrence and development of SLE. Although it is difficult to speculate as to how the members of SLE-associated virome species contribute to the occurrence and development of this disease, given that disease progression could be affected directly and indirectly by their relationships with the virome and human host physiology, development, and immunity, it is interesting for SLE researchers to explore the virome-mediated virulence mechanisms.

In conclusion, we did a metatranscriptional analysis to determine the global taxonomic distribution of the virome community composition in PBMCs from SLE patients and HCs. Moreover, we identified a minimal set of 30 viral species and 30 viral genera that could best distinguish SLE patients from HCs. However, due to the limited sample size and the exploratory nature of the transcriptomic sequencing, the claim that some viruses can be useful as diagnostic markers for SLE is rather preliminary and requires further validation, using more samples for NGS-seq analyses. Furthermore, we detected that this phenomenon only occurs within individual genes of some specific viruses (a paired-end read uniquely mapped with one end to hg19 and with the other end to the viruses' genome), which indicated that individual viral genes are integrated into the host genome. Nevertheless, further analyses showed that other genes of these specific viruses might also be transcribed in the same disease condition. In this study, we considered the viruses' overall abundance as a proxy of the viruses' ability to be a diagnostic marker of systemic lupus erythematosus. Therefore, although a possible bias due to the integration phenomenon of individual genes might occur with individual viruses, this drawback does not affect the diagnostic power of the viruses' overall abundance as a diagnostic biomarker of systemic lupus erythematosus described in this study. Although a small number of integrated genes are actively transcribed, the integration of viral genes is a phenomenon that warrants further studies at the DNA level to comprehensively analyze the correlation of viral integration and respective gene expression levels. Continued efforts for multi-kingdom profiling, coupled with functional studies of the RNA virome in PBMCs from human SLE patients, are needed to obtain new insights into the mechanisms through which the virome “dark matter” of the human host immunity contributes to the development and progression of SLE. Furthermore, we could uncover the diversity of the PBMC DNA virome in SLE, which would be focused upon in future studies.

## Data Availability Statement

The raw data supporting the conclusions of this article will be made available by the authors, without undue reservation, to any qualified researcher.

## Ethics Statement

The research protocol was approved by the Medical Ethical Committees of the First and Second Affiliated Hospitals of Wenzhou Medical University. All subjects who participated in this research provided written informed consent.

## Author Contributions

GG and LY performed the experiments, analyzed and interpreted the data, statistical analysis, and drafted the manuscript. XS collected samples and performed the experiments. KY, JH, KL, DX, SY, YW, and BL acquired the data and provided material support. CC acquired the data, provided material support and provided the project funding. XX and HZ contributed to the conception and design of the study, analyzed and interpreted the data, supervised the study, provided the project funding, revised the manuscript, and finally approved the version of the manuscript for publication. All authors read and approved the final manuscript.

### Conflict of Interest

The authors declare that the research was conducted in the absence of any commercial or financial relationships that could be construed as a potential conflict of interest.

## Supplementary Material

Supplementary Figure 1 Detection of integrated virus sequences in the whole-genome sequencing data sets using bioinformatic analysis. The flowchart summarizes the steps for identifying the viral sequences in peripheral blood mononuclear cells (PBMCs) of systemic lupus erythematosus (SLE) and healthy control (HCs) subjects.

Supplementary Figure 2 Diagnostic value of virome markers for systemic lupus erythematosus (SLE). Internal 10-times repeated 10-fold cross-validations of SLE species- (upper panel), genus- (middle panel), and host-based (lower panel) metatranscriptomic classifier in high (left panel) and low (right panel) abundance viral groups of SLE patients and in viral group of HCs. Red line represents the average true and false positive rates. Out dots depict each individual round of tenfold model cross-validations, which is summarized by boxplots.

Supplementary Figure 3 Disease-associated dysbiosis of the peripheral blood mononuclear cell (PBMC) virome in systemic lupus erythematosus (SLE) patients. Biplot showing consistent separation at species **(A)** and genus **(B)** levels, but not at the host level **(C)**. Projection axes were assessed individually by Wilcoxon's rank-sum tests. NS, No significant difference; ^*^*P* < 0.05; ^***^*P* < 0.001.

Supplementary Figure 4 Potential of peripheral blood mononuclear cell (PBMC) virome taxa for diagnosis of systemic lupus erythematosus (SLE) at different levels of species **(A)**, genus **(B)**, and host **(C)**, analyzed and evaluated as described for Figure 4.

Supplementary Figure 5 Gene ontology (GO) processes and Analyze Network (AN) analyses of candidate peripheral blood mononuclear cell (PBMC) viral markers associated differentially expressed genes (DEGs) in systemic lupus erythematosus (SLE). **(A)** GO analysis: cellular processes. Sorting is according to “Statistically significant processes.” **(B)** AN top-scored pathways (by the number of pathways). Thick cyan lines indicate fragments of canonical pathways. Upregulated and downregulated genes are indicated by red and blue circles, respectively. The “checkerboard” pattern indicates mixed expression. **(C)** PCR detection and validation of sequencing results for the selected viral targets. Gels were run to test for the presence of fragments from three bacterial viruses (NC_011356.1, 97bp; NC_019716.1, 101bp; NC_003287.2, 125bp) in PBMCs of five mixed SLE patients and five mixed HCs. M: 100-bp DNA marker; (1) Mixed templates from five additional samples of PBMCs from SLE patients; (2) Mixed templates from five additional samples of PBMCs from HCs; (3) NC1 (negative control 1): no template control from reverse transcription (RNA); (4) NC2 (negative control 2): water, no template control from PCR detection; PCR assays were performed using cDNA reverse transcribed from RNA extracted from PBMCs and suspended in 25 μL of DNase/RNase-free water. The reverse transcription and PCR experiments were performed in different facilities. No-template controls were included to confirm that the PCR reagents were included in every experiment. Moreover, sequencing was used to further confirm the specificity of PCR detection.

Supplementary Table 1 Detected viruses in PBMCs of SLE patients and Normal controls.

Supplementary Table 2 Detected viruses characteristic (except phage) in PBMCs of SLE patients and Normal controls.

Supplementary Table 3 Summary of Spearman rank Correlation Analysis of Human DEGs and candicate marker viruses.

Supplementary Table 4 Annotation of candicate viruses marker associated DEGs in SLE by KEGG and GO terms.

Supplementary Table 5 Most relevant biological networks using Analyze Networks (AN) algorithm with default settings.
