# Supplementary figures and images for: Dysbiosis in Peripheral Blood Mononuclear Cell Virome Associated With Systemic Lupus Erythematosus
Source: Front Cell Infect Microbiol. 2020 Apr 6;10:131. doi: 10.3389/fcimb.2020.00131 (PMC7153479; doi:10.3389/fcimb.2020.00131)

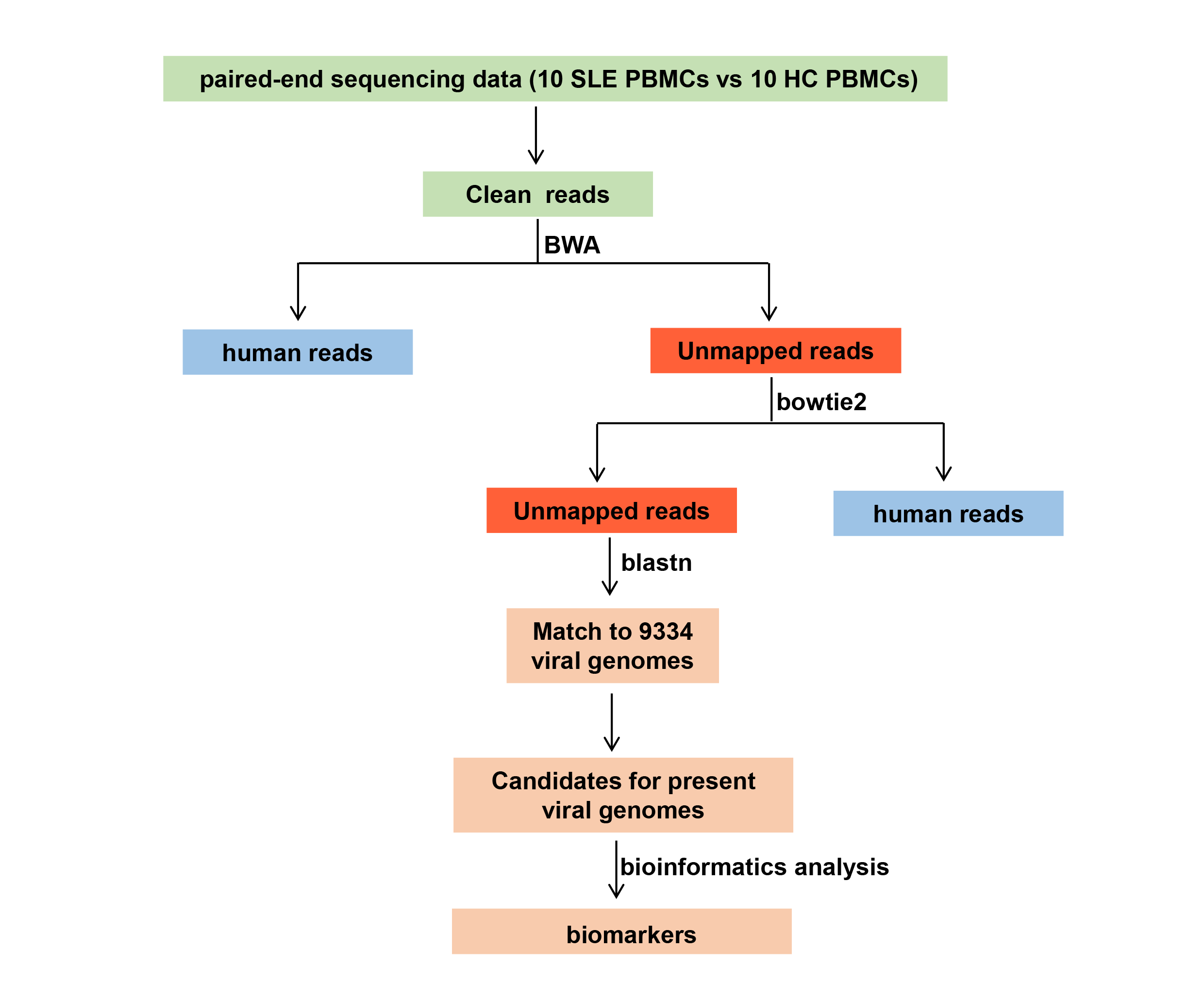

Supplement: Supplementary Figure 1 — Detection of integrated virus sequences in the whole-genome sequencing data sets using bioinformatic analysis. The flowchart summarizes the steps for identifying the viral sequences in peripheral blood mononuclear cells (PBMCs) of systemic lupus erythematosus (SLE) and healthy control (HCs) subjects. [file Presentation_1.zip › Supplementary Figure 1.TIF]

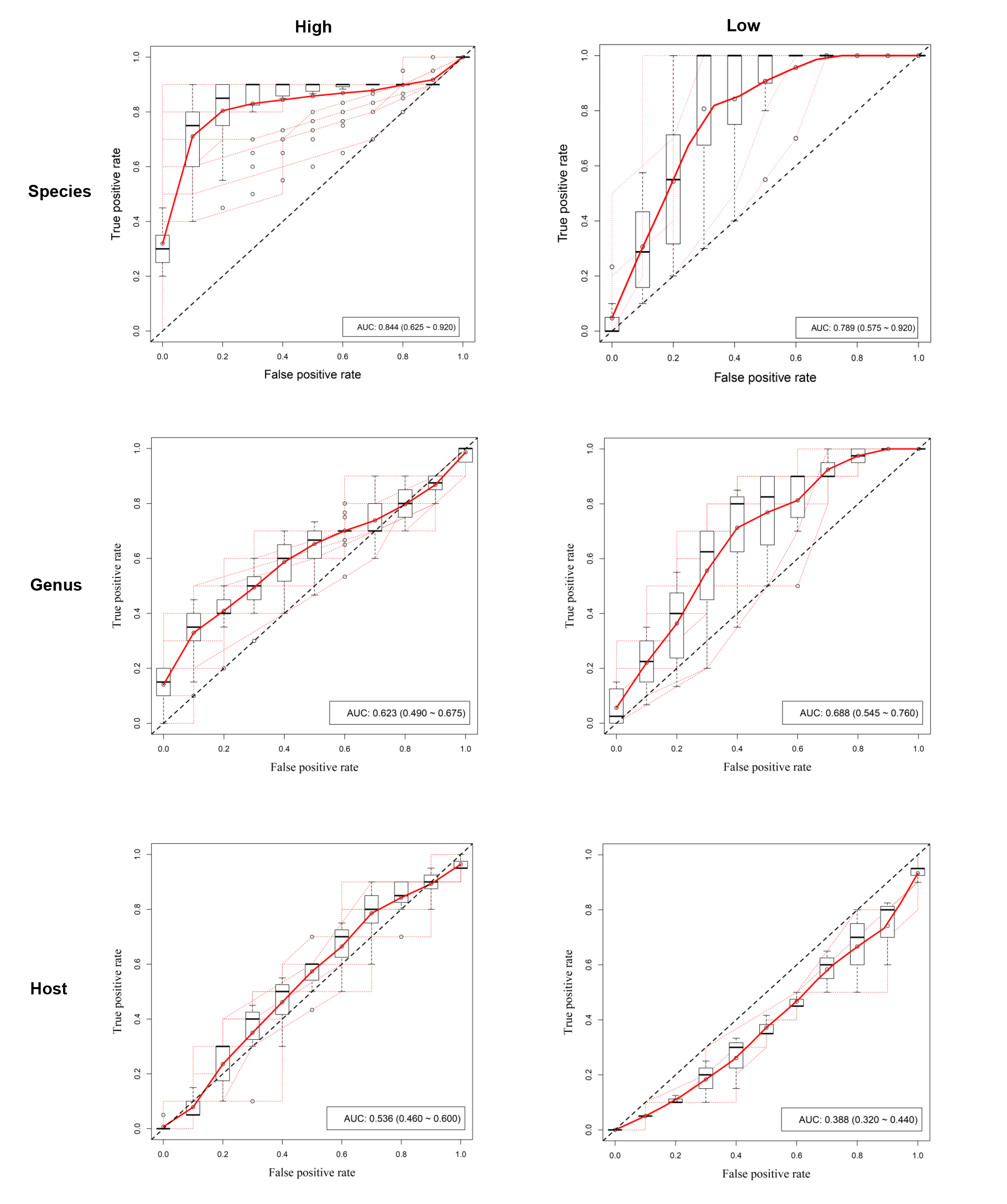

Supplement: Supplementary Figure 1 — Detection of integrated virus sequences in the whole-genome sequencing data sets using bioinformatic analysis. The flowchart summarizes the steps for identifying the viral sequences in peripheral blood mononuclear cells (PBMCs) of systemic lupus erythematosus (SLE) and healthy control (HCs) subjects. [file Presentation_1.zip › Supplementary Figure 2.TIF]

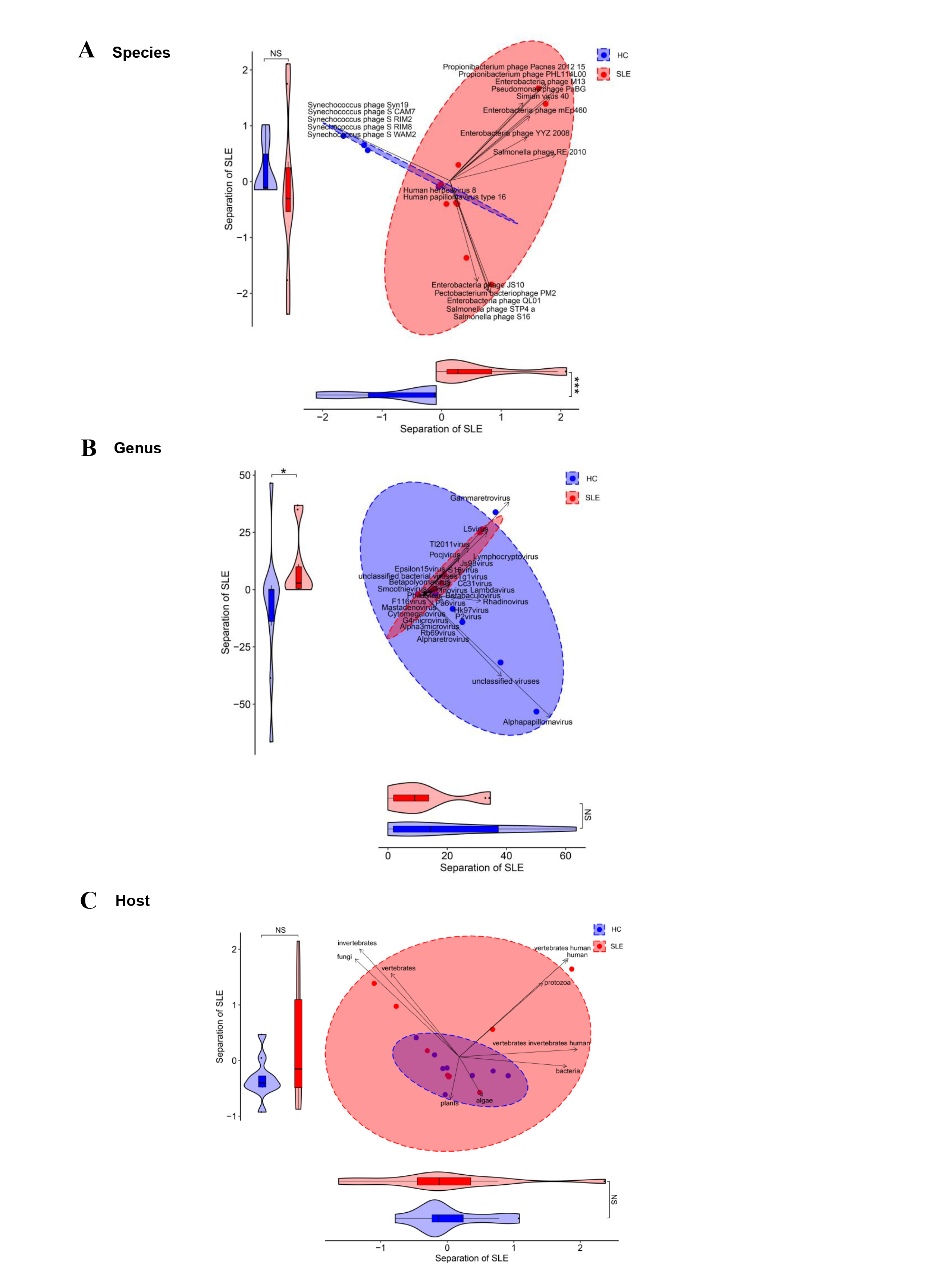

Supplement: Supplementary Figure 1 — Detection of integrated virus sequences in the whole-genome sequencing data sets using bioinformatic analysis. The flowchart summarizes the steps for identifying the viral sequences in peripheral blood mononuclear cells (PBMCs) of systemic lupus erythematosus (SLE) and healthy control (HCs) subjects. [file Presentation_1.zip › Supplementary Figure 3.TIF]

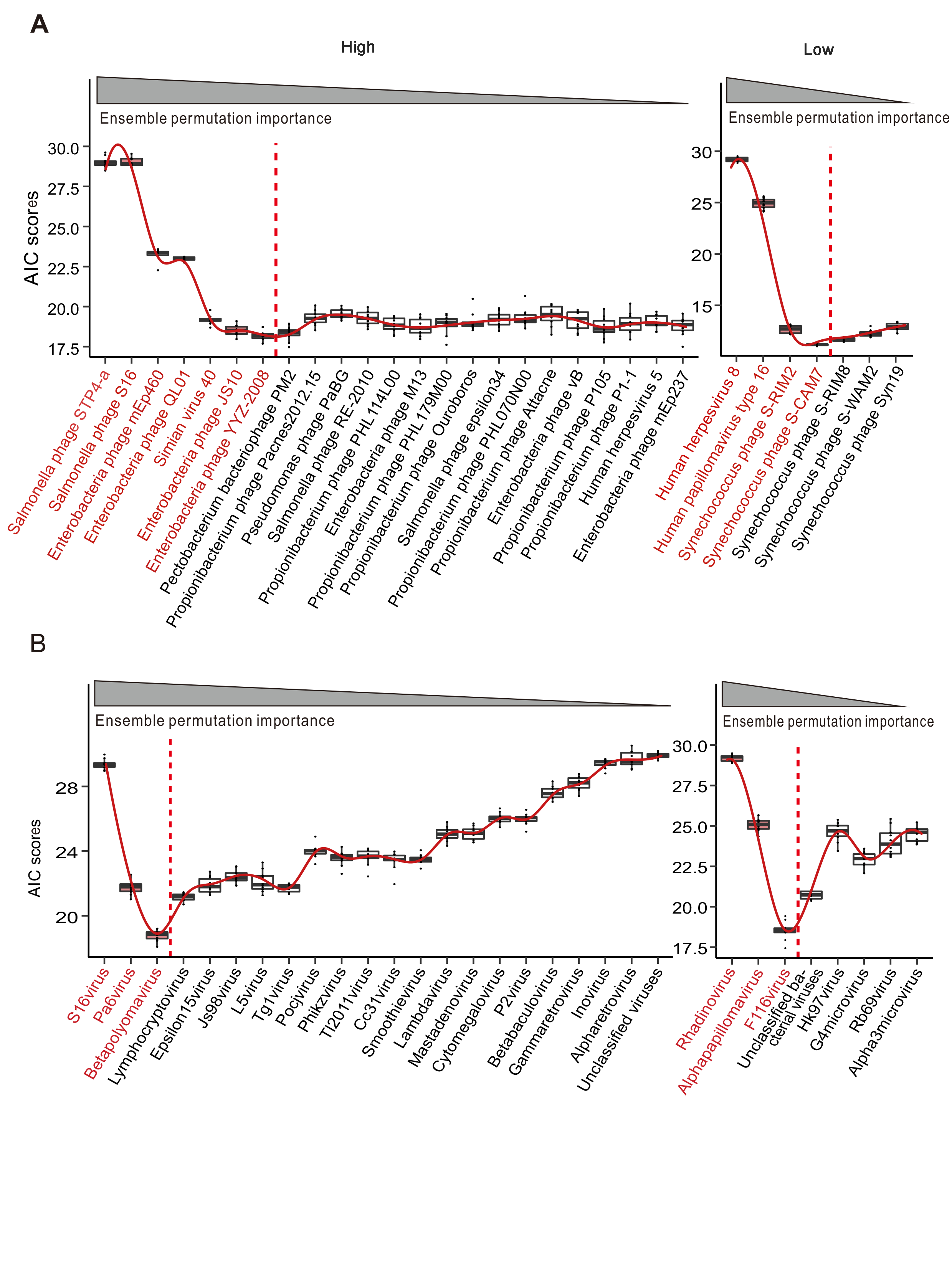

Supplement: Supplementary Figure 1 — Detection of integrated virus sequences in the whole-genome sequencing data sets using bioinformatic analysis. The flowchart summarizes the steps for identifying the viral sequences in peripheral blood mononuclear cells (PBMCs) of systemic lupus erythematosus (SLE) and healthy control (HCs) subjects. [file Presentation_1.zip › Supplementary Figure 4A,B.TIF]

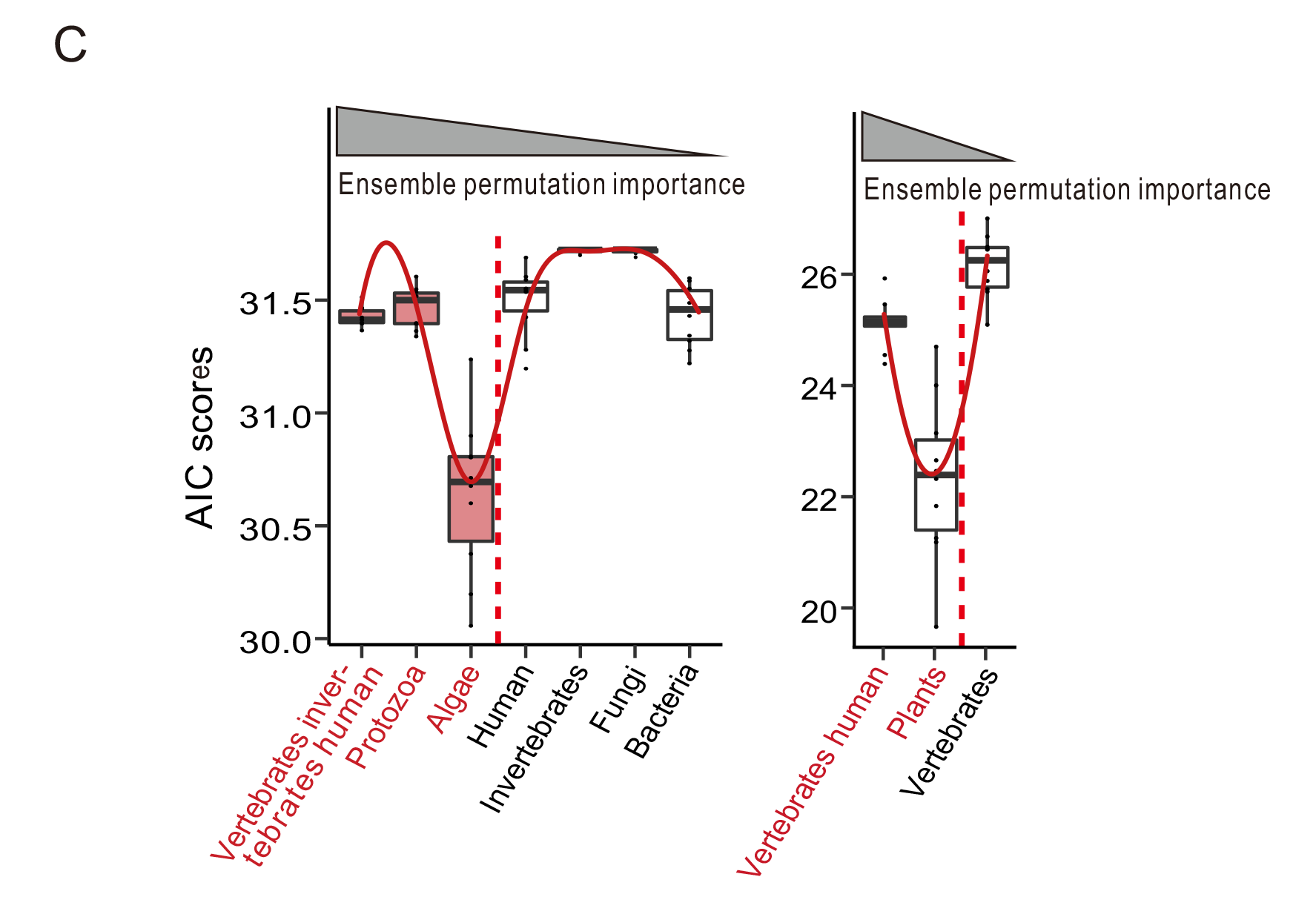

Supplement: Supplementary Figure 1 — Detection of integrated virus sequences in the whole-genome sequencing data sets using bioinformatic analysis. The flowchart summarizes the steps for identifying the viral sequences in peripheral blood mononuclear cells (PBMCs) of systemic lupus erythematosus (SLE) and healthy control (HCs) subjects. [file Presentation_1.zip › Supplementary Figure 4C.TIF]

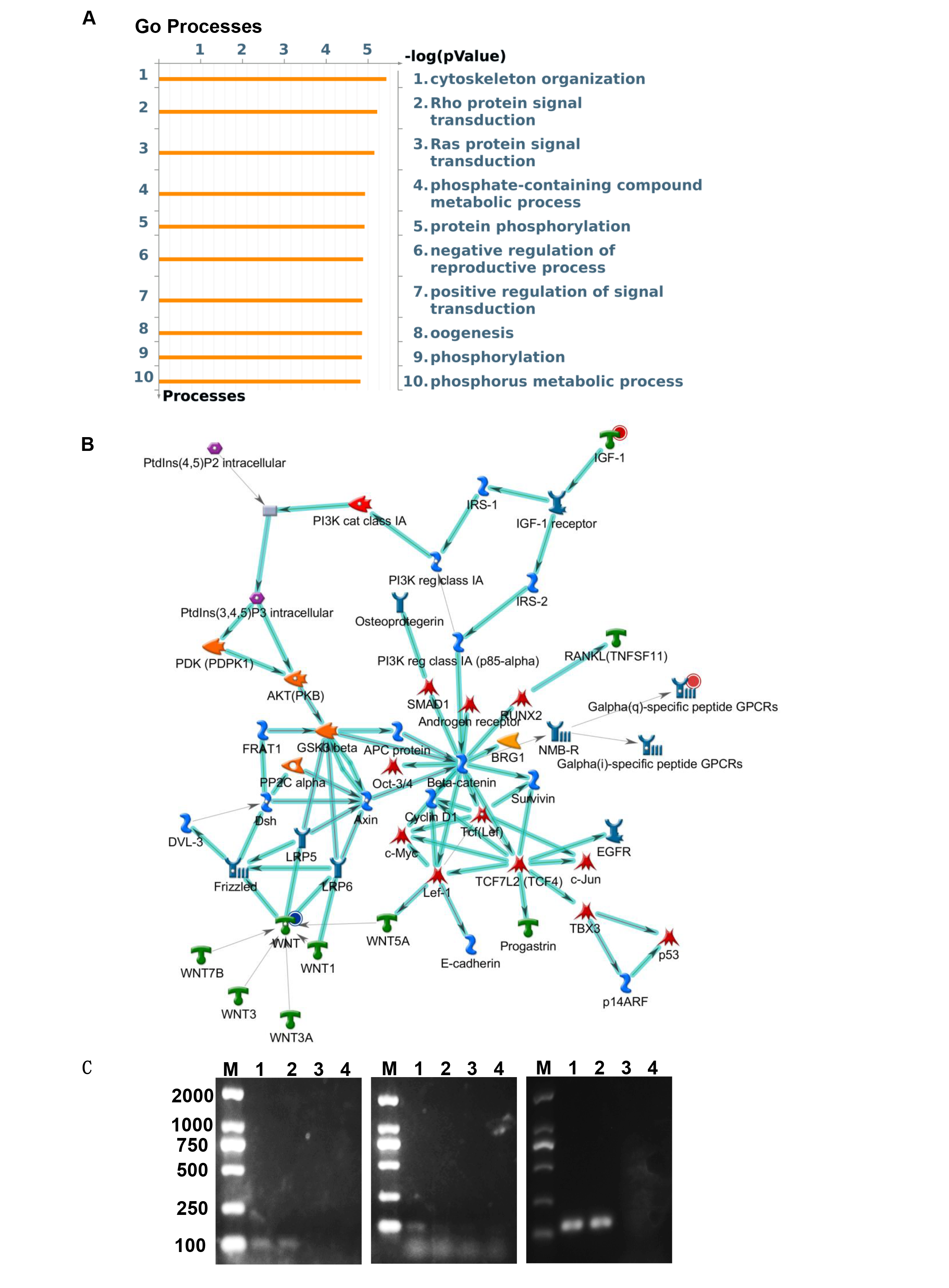

Supplement: Supplementary Figure 1 — Detection of integrated virus sequences in the whole-genome sequencing data sets using bioinformatic analysis. The flowchart summarizes the steps for identifying the viral sequences in peripheral blood mononuclear cells (PBMCs) of systemic lupus erythematosus (SLE) and healthy control (HCs) subjects. [file Presentation_1.zip › Supplementary Figure 5.TIF]
