# Supplementary material for: Dysbiosis in Peripheral Blood Mononuclear Cell Virome Associated With Systemic Lupus Erythematosus
Source: Front Cell Infect Microbiol. 2020 Apr 6;10:131. doi: 10.3389/fcimb.2020.00131 (PMC7153479; doi:10.3389/fcimb.2020.00131)
Supplement: Supplementary file 3 [file Data_Sheet_2.docx]

**Details of the bioinformatic analysis code and description in this study can be found below.**

##Set working directory according to the filepath where data files are stored

setwd()

getwd()

###Load packages######

library(VennDiagram)

library(vegan)

library(ggplot2)

library(ggpubr)

library(tidyverse)

require(randomForest)

library(affy)

library(gplots)

library(ROCR)

library(ggvegan)

library(FactoMineR)

library(ggord)

library(ggrepel)

library(plyr)

source('geom_ord_ellipse.R')

library(ca)

library(Hmisc)

library(pheatmap)

library("reshape2")

###Read data########

###Distribution of PBMCs virus species in species, genus and host levels###

Dat_species = read.csv("Species_Fig1_venn.csv")

probe1 = Dat_species$HC

probe2 = Dat_species$SLE

#Takes a list and creates a TIFF Venn Diagram

venn.diagram(list("HC"=probe1,"SLE"=probe2),cex=6,height=4800,width=4800,resolution=600,imagetype="tiff",cat.just = list(c(0, 0.8), c(1,0.8)), filename="Species_Fig1_venn.tiff", cat.fontface=15,na = "remove", fill = c("cornflowerblue","pink"),cat.cex =7, label.col="black", cat.col = c("cornflowerblue","pink"))

#Acquire the common species

probes3 = intersect(probe1,probe2)

write.table(probes3,"Species_Fig1_venn_intersection.txt",col.names = T,row.names = F,quote=F,sep="\t")

Dat_genus = read.csv("Genus_Fig1_venn.csv")

probe4 = Dat_genus$HC

probe5 = Dat_genus$SLE

venn.diagram(list("HC"=probe4,"SLE"=probe5),cex=6,height=4800,width=4800,resolution=600,imagetype="tiff",cat.just = list(c(0, 0.8), c(1, 0.8)),filename="Genus_Fig1_venn.tiff", cat.fontface=15,na = "remove", fill = c("cornflowerblue","pink"),cat.cex =7,label.col="black",cat.col = c("cornflowerblue","pink"))

probes6 = intersect(probe4,probe5)

write.table(probes6,"Genus_Fig1_venn_intersection.txt",col.names = T,row.names = F,quote=F,sep="\t")

Dat_host = read.csv("Host_Fig1_venn.csv")

probe7 = Dat_host$HC

probe8= Dat_host$SLE

venn.diagram(list("HC"=probe7,"SLE"=probe8),cex=6,height=4800,width=4800,resolution=600,imagetype="tiff",cat.just = list(c(0, 0.8), c(1, 0.8)),filename="Host_Fig1_venn.tiff", cat.fontface=15,na = "remove", fill = c("cornflowerblue","pink"),cat.cex =7,label.col="black", cat.col = c("cornflowerblue","pink"))

probes9 = intersect(probe7,probe8)

write.table(probes9,"Host_Fig1_venn_intersection.txt",col.names = T,row.names = F,quote=F,sep="\t")

###Evaluation of PBMCs virus species richness###

Dat_HC_419 <- read.csv("419_N.csv")

rownames(Dat_HC_419) <- Dat_HC_419[,1]

Dat_HC_419 <- Dat_HC_419[,-1]

Dat_SLE_419 <- read.csv("419_S.csv")

rownames(Dat_SLE_419) <- Dat_SLE_419[,1]

Dat_SLE_419 <- Dat_SLE_419[,-1]

#Find species accumulation curves or the number of species for a certain number of individuals.

sp1 <- specaccum(Dat_HC_419)

sp2 <- specaccum(Dat_HC_419,"rarefaction")

sp3 <- specaccum(Dat_SLE_419)

sp4 <- specaccum(Dat_SLE_419,"rarefaction")

SLE_rarefaction <-data.frame(cbind(sp4$sites,sp4$individuals,sp4$richness,sp4$sd))

HC_rarefaction <-data.frame(cbind(sp2$sites,sp2$individuals,sp2$richness,sp2$sd))

write.csv(SLE_rarefaction,"sle.csv")

write.csv(HC_rarefaction,"hc.csv")

###Graph the species diversity of PBMCs viromes###

Dat_virus_species <- read.csv("Fig1B.csv")

Dat_virus_species <- Dat_virus_species[1:20,]

dodge <- position_dodge(width = 0.1)

ggplot(data = Dat_virus_species, aes(x =Virus,y = number,color=Condition)) +geom_point(size=5)+geom_line(size=1.3) + scale_fill_discrete(labels=c("Health","SLE"))+ scale_color_manual(values = alpha(c("blue", "red"), .6))+ scale_x_continuous(breaks=seq(0, 10, 1))+xlab("") + ylab("Virus species") +ggtitle("")+theme_bw() + theme(plot.title = element_text(hjust = 0.5,size=26),panel.border = element_blank(), panel.grid.major = element_blank(),panel.grid.minor = element_blank(), axis.line = element_line(colour = "black"),axis.title.x=element_text(size=24),axis.title.y=element_text(size=24,vjust = 3), axis.text=element_text(colour="black",size=22),legend.position="top",legend.title = element_text(size=20),legend.text = element_text(size=19))

### Graph the abundance diversity of PBMCs viromes###

Dat_virus_richness <- read.csv("Fig1D_1.csv")

dodge <- position_dodge(width = 1.0)

compare_means(Abundance~Condition, data = Dat_virus_richness, method = "wilcox.test")

ggplot(data = Dat_virus_richness, aes(x =Condition,y = Abundance,fill=Condition))+ scale_fill_manual(values = alpha(c("blue", "red"), 1))+scale_color_manual(values = c("steelblue","darkred"))+geom_boxplot(width=0.5,outlier.colour = NA, position = dodge,notch = TRUE)+ xlab("") + ylab("Virus reads richness") + ggtitle("")+ylim(0,0.020)+ theme_bw() + theme(plot.title = element_text(size=26,hjust = 0.5,face="plain"),panel.border = element_blank(), panel.grid = element_blank(),axis.line = element_line(colour = "black"), axis.title=element_text(size=24,face="plain"),axis.text=element_text(colour="black",size=22,hjust=0.5,face="plain"),axis.ticks.x = element_blank(),legend.title = element_text(size = 20),legend.text = element_text(size=19),legend.position = "top")+stat_compare_means(label = "p.signif",label.y = 0.025,hjust=-1.3,method = "wilcox.test",size = 10)

### Graph the order distribution of PBMCs viromes###

Dat_order_proportion <- read.csv("le.csv")

link_dat <- Dat_order_proportion %>%

arrange(by=desc(Genus)) %>%

mutate(HC=cumsum(HC), SLE=cumsum(SLE))

Dat_order_proportion.long <- df %>% gather(Condition, Abundance, -Genus)

##Also can code like this: Dat_order_proportion.long <- reshape2::melt(Dat_order_proportion, value.name='Abundance', variable.name='Condition')

ggplot(Dat_order_proportion.long, aes(x=Condition, y=Abundance, fill=Genus)) + geom_bar(stat = "identity", width=0.5, col='black') + geom_segment(data=link_dat, aes(x=1.25, xend=1.75, y=HC, yend=SLE))+geom_text(mapping = aes(label = Genus),size = 5, colour = 'black', vjust = 3.5, hjust = .5, position = position_stack())

###Selection and evaluation of important species, genus and host###

Dat_species_MDA <- read.csv("20181019_dat7_randomForest.csv")

#Based on Breiman and Cutler’s original Fortran code to implement Breiman’s random forest algorithm for classification and regression.

MDA_species <- randomForest(condition ~ ., data= Dat_species_MDA, ntree=1000, keep.forest=FALSE,nperm = 10, cv.fold=10,importance=TRUE)

varImpPlot(MDA_species) #Dotchart of variable importance as measured by a Random Forest

write.csv(importance(MDA_species),file='MDA_species.csv',row.name=TRUE) #The class-specific measures computed as mean descrease in accuracy

OOB_species <-as.data.frame(MDA_species$err.rate) #vector error rates of the prediction on the input data,

write.csv(OOB_species,"OOB_Species.csv")

Dat_genus_MDA <- read.csv("RRHG_Genus_abundance_MDA_input.csv")

MDA_genus <- randomForest(Condition ~ ., data= Dat_genus_MDA, ntree=1000, keep.forest=FALSE,nperm = 10, cv.fold=10,importance=TRUE)

varImpPlot(MDA_genus)

write.csv(importance(MDA_genus),file='MDA_Genus.csv',row.name=TRUE)

OOB_genus <-as.data.frame(MDA_genus$err.rate)

write.csv(OOB_genus,"OOB_Genus.CSV")

Dat_host_MDA <- read.csv("RRHG_NCBI_Host_abundance_MDA_input.csv")

MDA_host <- randomForest(Condition ~ ., data= Dat_host_MDA, ntree=1000, keep.forest=FALSE,nperm = 10, cv.fold=10,importance=TRUE)

varImpPlot(MDA_host)

write.csv(importance(MDA_host),file='MDA_Host.csv',row.name=TRUE)

OOB_host <-as.data.frame(MDA_host $err.rate)

write.csv(OOB_host,"OOB_Host.CSV")

###Graph of important species abundance in species, genus and host levels###

MDA_species_heatmap = read.csv("20181019_dat8_heatmap.csv")

rownames(MDA_species_heatmap) = MDA_species_heatmap[,1]

MDA_species_heatmap = MDA_species_heatmap[,-1]

deg_species = read.table("SLE_Health_MDA.txt",header=T,sep="\t")

deg_species1 = MDA_species_heatmap[match(deg_species$virus,rownames(MDA_species_heatmap)), ]

lmat <- rbind( c(4,3,0), c(2,1,0) )

lhei <- c(1.0, 4)

lwid <- c(1.0, 4, 2.3)

heatmap.2(as.matrix(deg_species1), col = greenred(75), scale = "row", dendrogram = 'none', Colv=FALSE,Rowv=FALSE, key = TRUE, symkey = FALSE, density.info = "none", trace = "none", cexRow = 1.6 ,cexCol = 1.6, srtCol=0, adjCol = c(0.5,1), lmat=lmat,lhei=lhei, lwid=lwid, colsep, sepcolor="white", sepwidth=c(0.05,0.05), offsetRow = 0.1,offsetCol = 0.1, main = "")

deg_genus = read.table("RRHG_Genus_abundance_MDA.txt",header=T,sep="\t",row.names = 1)

heatmap.2(as.matrix(deg_genus), col = greenred(75), scale = "row", dendrogram = 'none', Colv=F,Rowv=FALSE, key = TRUE, symkey = FALSE, density.info = "none", trace = "none", cexRow = 1.6 ,cexCol = 1.6, srtCol=0, adjCol = c(0.5,1), lmat=lmat,lhei=lhei, lwid=lwid,family="TNR", offsetRow = 0.1,offsetCol = 0.1, main = "")

deg_host = read.table("RRHG_Host_abundance_MDA.txt",header=T,sep="\t",row.names = 1)

heatmap.2(as.matrix(deg_host), col = greenred(75), scale = "row", dendrogram = 'none', Colv=F,Rowv=FALSE, key = TRUE, symkey = FALSE, density.info = "none", trace = "none", cexRow = 1.6 ,cexCol = 1.6, srtCol=0, adjCol = c(0.5,1), lmat=lmat,lhei=lhei, lwid=lwid, offsetRow = 0.1,offsetCol = 0.1, main = "")

###Judgment ability of above important virus in species, genus and host levels###

MDA_species_roc <- read.csv('20181019_dat9_1_MDA_data1.csv')

Result_MDA_species_roc <- rfcv(MDA_species_roc[1:30], MDA_species_roc$condition, cv.fold=10,scale="log", step=0.99) #Get the cross-validated prediction performance of models with sequentially reduced number of predictors via a nested cross-validation procedure

Result_MDA_species_roc_2 <- Result_MDA_species_roc$predicted #The predicted values from the crossvalidation

Result_MDA_species_roc_5<-read.csv("roc.csv")

Result_MDA_species_roc_6<-as.list(Result_MDA_species_roc_5)

Result_MDA_species_roc_3 <- list(predictions = Result_MDA_species_roc_2, labels = Result_MDA_species_roc_6)

#Transform the input data into a standardized format to creat a prediction object.

pred.svm <- prediction(Result_MDA_species_roc_3$predictions, Result_MDA_species_roc_3$labels)

perf.svm <- performance(pred.svm,'auc') #Perform 'auc' predictor evaluations

auc_min<- min(as.numeric(as.data.frame(perf.svm@y.values)))

auc_max<- max(as.numeric(as.data.frame(perf.svm@y.values)))

auc_mean<- mean(as.numeric(as.data.frame(perf.svm@y.values)))

auc<-as.character(round(c(auc_mean, auc_min, auc_max),3))

auc

perf.svm <- performance(pred.svm,"tpr", "fpr") #Perform "tpr"&"fpr" predictor evaluations

plot(cex.axis=1.5, perf.svm,cex.lab=1.5,lty=3, col="red",main="")

plot(perf.svm, avg="vertical", lwd=3, col="red", spread.estimate='boxplot',plotCI.lwd=2,add=TRUE)

legend(0.6,0.05,c("AUC: 0.883 (0.635 ~ 0.950)"),text.col=c("black"),bty="O")

lines(par()$usr[1:2], par()$usr[3:4], lty=2, lwd=2, col="black")

MDA_species_roc_up <- read.csv('20181019_dat9_up23_MDA_data.csv')

Result_MDA_species_roc_up <- rfcv(MDA_species_roc_up[1:23], MDA_species_roc_up$condition, cv.fold=10,scale="log", step=0.99)

Result_MDA_species_roc_up_2 <- Result_MDA_species_roc_up$predicted

Result_MDA_species_roc_up_5 <- read.csv("roc.csv")

Result_MDA_species_roc_up_5 <- Result_MDA_species_roc_up_5[,1:23]

Result_MDA_species_roc_up_6 <- as.list(Result_MDA_species_roc_up_5)

Result_MDA_species_roc_up_3 <- list(predictions = Result_MDA_species_roc_up_2, labels = Result_MDA_species_roc_up_6)

pred.svm <- prediction(Result_MDA_species_roc_up_3$predictions, Result_MDA_species_roc_up_3$labels)

perf.svm <- performance(pred.svm,'auc')

auc_min<- min(as.numeric(as.data.frame(perf.svm@y.values)))

auc_max<- max(as.numeric(as.data.frame(perf.svm@y.values)))

auc_mean<- mean(as.numeric(as.data.frame(perf.svm@y.values)))

auc<-as.character(round(c(auc_mean,auc_min,auc_max),3))

auc

perf.svm <- performance(pred.svm,"tpr", "fpr")

plot(perf.svm,cex.lab=1.5,lty=3, col="red",main=" ")

plot(perf.svm, avg="vertical", lwd=3, col="red", spread.estimate='boxplot',plotCI.lwd=2,add=TRUE)

legend(0.6,0.05,c("AUC: 0.844 (0.625 ~ 0.920)"),text.col=c("black"),bty="O")

lines(par()$usr[1:2], par()$usr[3:4], lty=2, lwd=2, col="black")

MDA_species_roc_down <- read.csv('20181019_dat9_down7_MDA_data.csv')

Result_MDA_species_roc_down <- rfcv(MDA_species_roc_down[1:7], MDA_species_roc_down$condition, cv.fold=10,scale="log", step=0.99)

Result_MDA_species_roc_down_2 <- Result_MDA_species_roc_down$predicted

Result_MDA_species_roc_down_5 <- read.csv("roc.csv")

Result_MDA_species_roc_down_5 <- Result_MDA_species_roc_down_5[,1:7]

Result_MDA_species_roc_down_6 <- as.list(Result_MDA_species_roc_down_5)

Result_MDA_species_roc_down_3 <- list(predictions = Result_MDA_species_roc_down_2, labels = Result_MDA_species_roc_down_6)

pred.svm <- prediction(Result_MDA_species_roc_down_3$predictions, Result_MDA_species_roc_down_3$labels)

perf.svm <- performance(pred.svm,'auc')

auc_min<- min(as.numeric(as.data.frame(perf.svm@y.values)))

auc_max<- max(as.numeric(as.data.frame(perf.svm@y.values)))

auc_mean<- mean(as.numeric(as.data.frame(perf.svm@y.values)))

auc<-as.character(round(c(auc_mean,auc_min,auc_max),3))

auc

perf.svm <- performance(pred.svm,"tpr", "fpr")

plot(perf.svm,cex.lab=1.5,lty=3, col="blue",main=" ")

plot(perf.svm, avg="vertical", lwd=3, col="blue", spread.estimate='boxplot',plotCI.lwd=2,add=TRUE)

legend(0.6,0.05,c("AUC: 0.789 (0.575 ~ 0.910)"),text.col=c("black"),bty="O")

lines(par()$usr[1:2], par()$usr[3:4], lty=2, lwd=2, col="black")

MDA_genus_roc <- read.csv('RRHG_Genus_abundance_MDA_ROC.csv')

Result_MDA_genus_roc <- rfcv(MDA_genus_roc[1:30], MDA_genus_roc$condition, cv.fold=10,scale="log", step=0.99)

Result_MDA_genus_roc_2 <- Result_MDA_genus_roc$predicted

Result_MDA_genus_roc_5<-read.csv("roc.csv")

Result_MDA_genus_roc_6<-as.list(Result_MDA_genus_roc_5)

Result_MDA_genus_roc_3 <- list(predictions = Result_MDA_genus_roc_2, labels = Result_MDA_genus_roc_6)

pred.svm <- prediction(Result_MDA_genus_roc_3$predictions, Result_MDA_genus_roc_3$labels)

perf.svm <- performance(pred.svm,'auc')

auc_min<- min(as.numeric(as.data.frame(perf.svm@y.values)))

auc_max<- max(as.numeric(as.data.frame(perf.svm@y.values)))

auc_mean<- mean(as.numeric(as.data.frame(perf.svm@y.values)))

auc<-as.character(round(c(auc_mean,auc_min,auc_max),3))

auc

perf.svm <- performance(pred.svm,"tpr", "fpr")

plot(perf.svm,cex.sub=2.5,cex.lab=1.5,lty=3, col="red", main="")

plot(perf.svm, avg="vertical", lwd=3, col="red", spread.estimate='boxplot',plotCI.lwd=2,add=TRUE)

legend(0.55,0.08,c("AUC: 0.695 (0.590 ~ 0.770)"),text.col=c("black"),cex=1.2,bty="O")

lines(par()$usr[1:2], par()$usr[3:4], lty=2, lwd=2, col="black")

MDA_genus_roc_up <- read.csv('RRHG_Genus_abundance_MDA_ROC_Up.csv')

Result_MDA_genus_roc_up <- rfcv(MDA_genus_roc_up[1:22], MDA_genus_roc_up$condition, cv.fold=10,scale="log", step=0.99)

Result_MDA_genus_roc_up_2 <- Result_MDA_genus_roc_up$predicted

Result_MDA_genus_roc_up_5 <- read.csv("roc.csv")

Result_MDA_genus_roc_up_5 <- Result_MDA_genus_roc_up_5[,1:22]

Result_MDA_genus_roc_up_6 <- as.list(Result_MDA_genus_roc_up_5)

Result_MDA_genus_roc_up_3 <- list(predictions = Result_MDA_genus_roc_up_2, labels = Result_MDA_genus_roc_up_6)

pred.svm <- prediction(Result_MDA_genus_roc_up_3$predictions, Result_MDA_genus_roc_up_3$labels)

perf.svm <- performance(pred.svm,'auc')

auc_min<- min(as.numeric(as.data.frame(perf.svm@y.values)))

auc_max<- max(as.numeric(as.data.frame(perf.svm@y.values)))

auc_mean<- mean(as.numeric(as.data.frame(perf.svm@y.values)))

auc<-as.character(round(c(auc_mean,auc_min,auc_max),3))

auc

perf.svm <- performance(pred.svm,"tpr", "fpr")

plot(perf.svm,cex.sub=2.5,cex.lab=1.5,lty=3, col="red", main="")

plot(perf.svm, avg="vertical", lwd=3, col="red", spread.estimate='boxplot',plotCI.lwd=2,add=TRUE)

legend(0.55,0.08,c("AUC: 0.623 (0.490 ~ 0.675)"),text.col=c("black"),cex=1.2,bty="O")

lines(par()$usr[1:2], par()$usr[3:4], lty=2, lwd=2, col="black")

MDA_genus_roc_down <- read.csv('RRHG_Genus_abundance_MDA_ROC_Down.csv')

Result_MDA_genus_roc_down <- rfcv(MDA_genus_roc_down[1:8], MDA_genus_roc_down$condition, cv.fold=10,scale="log", step=0.99)

Result_MDA_genus_roc_down_2 <- Result_MDA_genus_roc_down$predicted

Result_MDA_genus_roc_down_5 <- read.csv("roc.csv")

Result_MDA_genus_roc_down_5 <- Result_MDA_genus_roc_down_5[,1:8]

Result_MDA_genus_roc_down_6 <- as.list(Result_MDA_genus_roc_down_5)

Result_MDA_genus_roc_down_3 <- list(predictions = Result_MDA_genus_roc_down_2, labels = Result_MDA_genus_roc_down_6)

pred.svm <- prediction(Result_MDA_genus_roc_down_3$predictions, Result_MDA_genus_roc_down_3$labels)

perf.svm <- performance(pred.svm,'auc')

auc_min<- min(as.numeric(as.data.frame(perf.svm@y.values)))

auc_max<- max(as.numeric(as.data.frame(perf.svm@y.values)))

auc_mean<- mean(as.numeric(as.data.frame(perf.svm@y.values)))

auc<-as.character(round(c(auc_mean,auc_min,auc_max),3))

auc

perf.svm <- performance(pred.svm,"tpr", "fpr")

plot(perf.svm,cex.sub=2.5,cex.lab=1.5,lty=3, col="red", main="")

plot(perf.svm, avg="vertical", lwd=3, col="red", spread.estimate='boxplot',plotCI.lwd=2,add=TRUE)

legend(0.55,0.08,c("AUC: 0.688 (0.545 ~ 0.760)"),text.col=c("black"),cex=1.2,bty="O")

lines(par()$usr[1:2], par()$usr[3:4], lty=2, lwd=2, col="black")

MDA_host_roc <- read.csv('RRHG_Host_abundance_MDA_ROC.csv')

Result_MDA_host_roc <- rfcv(MDA_host_roc[1:10], MDA_host_roc$condition, cv.fold=10,scale="log", step=0.99)

Result_MDA_host_roc_2 <- Result_MDA_host_roc$predicted

Result_MDA_host_roc_5<-read.csv("roc.csv")

Result_MDA_host_roc_5<- Result_MDA_host_roc_5[,1:10]

Result_MDA_host_roc_6<-as.list(Result_MDA_host_roc_5)

Result_MDA_host_roc_3 <- list(predictions = Result_MDA_host_roc_2, labels = Result_MDA_host_roc_6)

pred.svm <- prediction(Result_MDA_host_roc_3$predictions, Result_MDA_host_roc_3$labels)

perf.svm <- performance(pred.svm,'auc')

auc_min<- min(as.numeric(as.data.frame(perf.svm@y.values)))

auc_max<- max(as.numeric(as.data.frame(perf.svm@y.values)))

auc_mean<- mean(as.numeric(as.data.frame(perf.svm@y.values)))

auc<-as.character(round(c(auc_mean,auc_min,auc_max),3))

auc

perf.svm <- performance(pred.svm,"tpr", "fpr")

plot(perf.svm,cex.sub=2.5,cex.lab=1.5,lty=3, col="red", main="")

plot(perf.svm, avg="vertical", lwd=3, col="red", spread.estimate='boxplot',plotCI.lwd=2,add=TRUE)

legend(0.55,0.08,c("AUC: 0.540 (0.400 ~ 0.630)"),text.col=c("black"),cex=1.2,bty="O")

lines(par()$usr[1:2], par()$usr[3:4], lty=2, lwd=2, col="black")

MDA_host_roc_up <- read.csv('RRHG_Host_abundance_MDA_ROC_Up.csv')

Result_MDA_genus_roc_up <- rfcv(MDA_host_roc_up[1:7], MDA_host_roc_up$condition, cv.fold=10,scale="log", step=0.99)

Result_MDA_genus_roc_up_2 <- Result_MDA_genus_roc_up$predicted

Result_MDA_genus_roc_up_5<-read.csv("roc.csv")

Result_MDA_genus_roc_up_5<- Result_MDA_genus_roc_up_5[,1:7]

Result_MDA_genus_roc_up_6<-as.list(Result_MDA_genus_roc_up_5)

Result_MDA_genus_roc_up_3 <- list(predictions = Result_MDA_genus_roc_up_2, labels = Result_MDA_genus_roc_up_6)

pred.svm <- prediction(Result_MDA_genus_roc_up_3$predictions, Result_MDA_genus_roc_up_3$labels)

perf.svm <- performance(pred.svm,'auc')

auc_min<- min(as.numeric(as.data.frame(perf.svm@y.values)))

auc_max<- max(as.numeric(as.data.frame(perf.svm@y.values)))

auc_mean<- mean(as.numeric(as.data.frame(perf.svm@y.values)))

auc<-as.character(round(c(auc_mean,auc_min,auc_max),3))

auc

perf.svm <- performance(pred.svm,"tpr", "fpr")

plot(perf.svm,cex.sub=2.5,cex.lab=1.5,lty=3, col="red", main="")

plot(perf.svm, avg="vertical", lwd=3, col="red", spread.estimate='boxplot',plotCI.lwd=2,add=TRUE)

legend(0.55,0.08,c("AUC: 0.536 (0.460 ~ 0.600)"),text.col=c("black"),cex=1.2,bty="O")

lines(par()$usr[1:2], par()$usr[3:4], lty=2, lwd=2, col="black")

MDA_host_roc_down <- read.csv('RRHG_Host_abundance_MDA_ROC_Down.csv')

Result_MDA_genus_roc_down <- rfcv(MDA_host_roc_down[1:3], MDA_host_roc_down$condition, cv.fold=10,scale="log", step=0.99)

Result_MDA_genus_roc_down_2 <- Result_MDA_genus_roc_down$predicted

Result_MDA_genus_roc_down_5 <- read.csv("roc.csv")

Result_MDA_genus_roc_down_5<- Result_MDA_genus_roc_down_5[,1:3]

Result_MDA_genus_roc_down_6<-as.list(Result_MDA_genus_roc_down_5)

Result_MDA_genus_roc_down_3 <- list(predictions = Result_MDA_genus_roc_down_2, labels = Result_MDA_genus_roc_down_6)

pred.svm <- prediction(Result_MDA_genus_roc_down_3$predictions, Result_MDA_genus_roc_down_3$labels)

perf.svm <- performance(pred.svm,'auc')

auc_min<- min(as.numeric(as.data.frame(perf.svm@y.values)))

auc_max<- max(as.numeric(as.data.frame(perf.svm@y.values)))

auc_mean<- mean(as.numeric(as.data.frame(perf.svm@y.values)))

auc<-as.character(round(c(auc_mean,auc_min,auc_max),3))

auc

perf.svm <- performance(pred.svm,"tpr", "fpr")

plot(perf.svm,cex.sub=2.5,cex.lab=1.5,lty=3, col="red", main="Virus host for prediction SLE (MDA&Down) in RRHG")

plot(perf.svm, avg="vertical", lwd=3, col="red", spread.estimate='boxplot',plotCI.lwd=2,add=TRUE)

legend(0.55,0.08,c("AUC: 0.388 (0.320 ~ 0.440)"),text.col=c("black"),cex=1.2,bty="O")

lines(par()$usr[1:2], par()$usr[3:4], lty=2, lwd=2, col="black")

###Ordination analysis###

P_species <- read.csv("20_species.csv")

rownames(P_species) = P_species[,1]

P_species <- P_species[,c(-1,-2)]

#Performs detrended correspondence analysis and basic reciprocal averaging or orthogonal correspondence analysis. If the first axis of lengths of gradient is greater than 4.0, choose CA; if it is between 3.0-4.0, choose RDA and CA; if it is less than 3.0, the result of RDA is better than CA.

decorana(P_species)

P_species_1=ca(P_species,scale = T) #Computation of simple correspondence analysis

write.csv(P_species_1$rowcoord, file = "20_species_output.csv",row.names = TRUE) #Get the row standard coordinates

P_genus <- read.csv("30_genus.csv")

rownames(P_genus) = P_genus[,1]

P_genus <- P_genus[,c(-1,-2)]

decorana(P_genus)

P_genus_1=ca(P_genus,scale = T)

write.csv(P_genus_1$rowcoord, file = "30_genus_output.csv",row.names = TRUE)

P_host <- read.csv("10_host.csv")

rownames(P_host) = P_host[,1]

P_host <- P_host[,c(-1,-2)]

decorana(P_host)

P_host_1=rda(P_host,scale = T)

write.csv(summary(P_host_1)$sites, file = "10_host_output.csv",row.names = TRUE)

#Graph of ordination analysis in species, genus and host levels

P_3species1 <- read.csv("20_species.csv")

P_3species1 <- within(P_3species1,{Condition3 <- factor(Condition3,levels=c('HC','Stable stage','Active stage'))})

P_3species1_scores <- read.csv("species_MDA20_output1.csv")

P_3species1_scores <- within(P_3species1_scores,{Condition3 <- factor(Condition3,levels=c('HC','Stable stage','Active stage'))})

P_3species3 <- P_3species1[,c(-1,-3)]

rownames(P_3species1) = P_3species1[,1]

P_3species2 <- P_3species1[,c(-1,-2,-3)]

ord <- PCA(P_3species3[,2:21])

P_3species <- ggord(ord, P_3species3$Condition3,cols = c("green",'blue','red'),obs.scale = 0, var.scale = 1, arrow=0.2,veclsz =0.2,vec_ext =8, size=2.5,scannf=TRUE,nfposi=3, txt=3, ext=1.1, nfac = 1,grp_title = "Condition") + theme_minimal() + theme(panel.grid = element_blank(), axis.text = element_blank(),axis.title = element_blank(), plot.title = element_blank(), legend.title = element_text(hjust =0.1,size=10), legend.text = element_text(size=9), legend.position="top", plot.margin = unit(c(0,-1,4,1),"cm")) + geom_ord_ellipse(ellipse_pro = .95, lty=5)

P_3species_comparisons <- list(c("HC", "Stable stage"), c("Stable stage", "Active stage"), c("HC", "Active stage"))

compare_means(Scores~Condition3,data= P_3species1_scores[c(-21:-40),],comparisons= P_3species_comparisons)

P_3species_x <- ggviolin(P_3species1_scores[c(-21:-40),], x="Condition3", y="Scores", fill="Condition3",palette = c("green","blue", "red"), trim =TRUE, position = position_dodge(0.3),alpha =0.3, add = c("boxplot"), ylab = "Separation of SLE", add.params=list(color='black',size=0.2)) + theme_bw()+rremove("legend")+rremove("grid") + rremove("y.axis")+ rremove("y.ticks")+rremove("y.text")+rremove("ylab") + theme(panel.border = element_blank(), axis.line = element_line(size=0.5, colour = "black")) + font("xlab",size = 12,color = "black") + font("x.text",size = 12,color = "black") + stat_compare_means(Scores~Condition3,data= P_3species1_scores[c(-21:-40),],label = "p.signif",vjust=5, comparisons= P_3species1_comparisons,size=5)+rotate()

compare_means(Scores~Condition3, data= P_3species1_scores[c(-1:-20),], comparisons= P_3species1_comparisons)

P_3species_y <- ggviolin(P_3species1_scores[c(-1:-20),], x="Condition3", y="Scores", fill="Condition3",palette = c("green","blue", "red"), position = position_dodge(0.3),alpha =0.3,trim =TRUE, add = c("boxplot"),ylab = "Separation of SLE", add.params=list(color= 'black',size=0.3)) + theme_bw()+rremove("legend")+rremove("grid")+ rremove("x.axis")+ rremove("x.ticks")+rremove("x.text")+rremove("xlab") + theme(panel.border = element_blank(), axis.line = element_line(size=0.5, colour = "black")) + font("ylab", size = 12,color = "black") + font("y.text",size = 12,color = "black") + stat_compare_means(label = "p.signif",vjust=1,hjust=-1,label.y = c(2.0, 2.3, 2.6), comparisons= P_3species1_comparisons, size = 5)

P_3species_x_grob <- ggplotGrob(P_3species_x)

P_3species_y_grob <- ggplotGrob(P_3species_y)

P_3species +annotation_custom(grob = P_3species_x_grob, xmin =-9, xmax =13, ymin = -16, ymax = -9.5) + annotation_custom(grob = P_3species_y_grob, xmin =-14, xmax =-7.5, ymin = -8, ymax = 10)

P_2species1 <- read.csv("20_species.csv")

P_2species1_scores <- read.csv("species_MDA20_output1.csv")

P_2species3<- P_2species1[,c(-1,-2)]

rownames(P_2species1)= P_2species1[,1]

P_2species2 <- P_2species1[,c(-1,-2,-3)]

ord <- PCA(P_2species3[,2:21])

P_2species <-ggord(ord, P_2species3$Condition2,cols = c('blue','red'),obs.scale = 0, var.scale = 1, arrow=0.2,veclsz =0.2,vec_ext =8, size=2.5,scannf=TRUE,nfposi=3, txt=3, ext=1.1, nfac = 1,grp_title = "Condition")+theme_minimal()+theme(panel.grid = element_blank(),axis.text = element_blank(),axis.title = element_blank(),plot.title = element_blank(),legend.title = element_text(hjust =0.1,size=10),legend.text = element_text(size=9), legend.position="top", plot.margin = unit(c(0,-1,4,1),"cm"))+geom_ord_ellipse(ellipse_pro = .95, lty=5)

compare_means(Scores~Condition2,data= P_2species1_scores[c(-21:-40),])

P_2species_x <- ggviolin(P_2species1_scores[c(-21:-40),], x="Condition2", y="Scores", fill= "Condition2",palette = c("blue", "red"), trim =TRUE,position = position_dodge(0.3),alpha =0.3, add = c("boxplot"), ylab = "Separation of SLE",add.params=list(color='black',size=0.2)) + theme_bw()+rremove("legend")+rremove("grid")+rremove("y.axis")+ rremove("y.ticks")+ rremove("y.text")+rremove("ylab")+ theme(panel.border = element_blank(),axis.line = element_line(size=0.5, colour = "black"))+font("xlab",size = 12,color = "black")+ font("x.text",size = 12,color = "black")+stat_compare_means(label = "p.signif", vjust=0, size=5,hjust=1)+rotate()

compare_means(Scores~Condition2, data= P_2species1_scores[c(-1:-20),])

P_2species_y <-ggviolin(P_2species1_scores[c(-1:-20),], x="Condition2", y="Scores",fill= "Condition2",palette = c("blue", "red"), position = position_dodge(0.3),alpha =0.3,trim = TRUE,add = c("boxplot"),ylab = "Separation of SLE", add.params = list(color='black',size =0.3))+theme_bw()+rremove("legend")+rremove("grid")+rremove("x.axis")+rremove("x.ticks")+rremove("x.text")+rremove("xlab")+theme(panel.border = element_blank(),axis.line = element_line(size=0.5, colour = "black"))+font("ylab", size = 12,color = "black")+ font("y.text",size = 12,color = "black")+stat_compare_means(label = "p.signif",vjust=1, hjust=0,label.y = c(2.0, 2.3, 2.6),size = 5)

P_2species_x_grob <- ggplotGrob(P_2species_x)

P_2species_y_grob <- ggplotGrob(P_2species_y)

P_2species +annotation_custom(grob = P_2species_x_grob, xmin =-8, xmax =7, ymin = -13.5, ymax = -9)+annotation_custom(grob = P_2species_y_grob, xmin =-12, xmax =-7.5, ymin = -8, ymax = 7)

P_3genus1 <-read.csv("30_genus.csv")

P_3genus1 <- within(P_3genus1,{Condition3<-factor(Condition3,levels=c('HC','Stable stage','Active stage'))})

P_3genus1_scores <- read.csv("genus1_PCA_data_output.csv")

P_3genus1_scores<-within(P_3genus1_scores,{Condition3<-factor(Condition3,levels=c('HC','Stable stage','Active stage')) })

P_3genus3<- P_3genus1[,c(-1,-3)]

rownames(P_3genus1)= P_3genus1[,1]

P_3genus2 <- P_3genus1[,c(-1,-2,-3)]

ord <- ca(P_3genus3[,2:31])

P_3genus <-ggord(ord, P_3genus3$Condition3,cols = c("green",'blue','red'),obs.scale = 0, var.scale = 1,arrow=0.2,veclsz =0.2,vec_ext =1,size=2.5,scannf=TRUE,nfposi=3,txt=3, ext=1.1,nfac = 1,grp_title = "Condition")+theme_minimal()+theme(panel.grid = element_blank(), axis.text = element_blank(),axis.title = element_blank(),plot.title = element_blank(),legend.title = element_text(hjust =0.1,size=10),legend.text = element_text(size=9),legend.position="top",plot.margin = unit(c(0.5,-2,6,1),"cm"))+ geom_ord_ellipse(ellipse_pro = .95, lty=5)

P_3genus1_comparisons <- list(c("HC", "Stable stage"), c("Stable stage", "Active stage"),c("HC", "Active stage"))

compare_means(Scores~Condition3,data= P_3genus1_scores[c(-21:-40),],comparisons= P_3genus1_comparisons)

P_3genus1_x <- ggviolin(P_3genus1_scores[c(-21:-40),], x="Condition3", y="Scores", fill="Condition3",palette = c("green","blue", "red"), trim =TRUE,position = position_dodge(0.3), alpha =0.3, add = c("boxplot"), ylab = "Separation of SLE", add.params=list(color='black',size=0.2))+theme_bw()+rremove("legend")+rremove("grid")+ rremove("y.axis")+ rremove("y.ticks")+rremove("y.text")+rremove("ylab")+ theme(panel.border = element_blank(),axis.line = element_line(size=0.5, colour = "black")) +font("xlab",size = 12,color = "black")+font("x.text",size = 12,color = "black")+ stat_compare_means(Scores~Condition3,data= P_3genus1_scores[c(-21:-40),],label = "p.signif",vjust=5,comparisons= P_3genus1_comparisons,size=5)+rotate()

compare_means(Scores~Condition3, data= P_3genus1_scores[c(-1:-20),], comparisons= P_3genus1_comparisons)

P_3genus1_y <-ggviolin(P_3genus1_scores[c(-1:-20),], x="Condition3", y="Scores", fill= "Condition3",palette = c("green","blue", "red"), position = position_dodge(0.3),alpha =0.3, trim =TRUE,add = c("boxplot"),ylab = "Separation of SLE", add.params = list(color='black', size=0.3))+theme_bw()+rremove("legend")+rremove("grid")+rremove("x.axis")+rremove("x.ticks")+rremove("x.text")+rremove("xlab")+theme(panel.border = element_blank(),axis.line = element_line(size=0.5, colour = "black"))+font("ylab", size = 12,color = "black")+ font("y.text",size = 12,color = "black")+stat_compare_means(label = "p.signif",vjust=1, hjust=-1,label.y = c(45, 50, 55),comparisons=P_3genus1_comparisons,size = 5)

P_3genus1_x_grob <- ggplotGrob(P_3genus1_x)

P_3genus1_y_grob <- ggplotGrob(P_3genus1_y)

P_3genus +annotation_custom(grob = P_3genus1_x_grob, xmin =-15, xmax =85, ymin = -110, ymax = -55)+annotation_custom(grob = P_3genus1_y_grob, xmin =-80, xmax =-25, ymin = -65, ymax = 53)

P_2genus1 <- read.csv("30_genus.csv")

P_2genus1_scores <- read.csv("genus1_PCA_data_output.csv")

P_2genus3<- P_2genus1[,c(-1,-2)]

rownames(P_2genus1)= P_2genus1[,1]

P_2genus2<- P_2genus1[,c(-1,-2,-3)]

ord <- ca(P_2genus3[,2:31])

P_2genus <-ggord(ord, P_2genus3$Condition2,cols = c('blue','red'),obs.scale = 0, var.scale = 1,arrow=0.2,veclsz =0.2,vec_ext =1,size=2.5,scannf=TRUE,nfposi=3,txt=3, ext=1.1,nfac = 1,grp_title = "Condition")+theme_minimal()+theme(panel.grid = element_blank(),axis.text = element_blank(),axis.title = element_blank(),plot.title = element_blank(),legend.title = element_text(hjust =0.1,size=10),legend.text = element_text(size=9),legend.position="top", plot.margin = unit(c(0.5,-2,6,1),"cm"))+geom_ord_ellipse(ellipse_pro = .95, lty=5)

compare_means(Scores~Condition2,data= P_2genus1_scores[c(-21:-40),])

P_2genus1_x <- ggviolin(P_2genus1_scores[c(-21:-40),], x="Condition2", y="Scores", fill= "Condition2",palette = c("blue", "red"), trim =TRUE,position = position_dodge(0.3), alpha =0.3, add = c("boxplot"), ylab = "Separation of SLE",add.params = list(color='black',size= 0.2))+theme_bw()+rremove("legend")+rremove("grid")+rremove("y.axis")+rremove("y.ticks")+rremove("y.text")+rremove("ylab")+theme(panel.border = element_blank(),axis.line = element_line(size=0.5, colour = "black"))+font("xlab",size = 12,color = "black")+ font("x.text",size = 12,color = "black")+stat_compare_means(label = "p.signif",vjust=-1.2,size=5,hjust=1)+rotate()

compare_means(Scores~Condition2, data= P_2genus1_scores[c(-1:-20),])

P_2genus1_y <-ggviolin(P_2genus1_scores[c(-1:-20),], x="Condition2", y="Scores", fill = "Condition2",palette = c("blue", "red"), position = position_dodge(0.3),alpha =0.3, trim= TRUE, add = c("boxplot"),ylab = "Separation of SLE",add.params = list(color='black',size =0.3))+theme_bw()+rremove("legend")+rremove("grid")+rremove("x.axis")+rremove("x.ticks")+rremove("x.text")+rremove("xlab")+theme(panel.border = element_blank(),axis.line = element_line(size=0.5, colour = "black"))+font("ylab", size = 12,color="black")+font("y.text", size = 12,color = "black")+stat_compare_means(label = "p.signif",vjust=1,hjust=-3,size = 5)

P_2genus1_x_grob <- ggplotGrob(P_2genus1_x)

P_2genus1_y_grob <- ggplotGrob(P_2genus1_y)

P_2genus + annotation_custom(grob = P_2genus1_x_grob, xmin =-20, xmax =65, ymin = -110, ymax = -70)+annotation_custom(grob = P_2genus1_y_grob, xmin =-80, xmax =-40, ymin = -65, ymax = 50)

P_3host1 <-read.csv("10_host.csv")

P_3host1 <- within(P_3host1,{Condition3<-factor(Condition3,levels=c('HC','Stable stage','Active stage'))})

P_3host1_scores <- read.csv("host1_PCA_data_output.csv")

P_3host1_scores<-within(P_3host1_scores,{Condition3<-factor(Condition3,levels=c('HC','Stable stage','Active stage'))})

P_3host3<- P_3host1[,c(-1,-3)]

rownames(P_3host1)= P_3host1[,1]

P_3host2 <- P_3host1[,c(-1,-2,-3)]

ord <- PCA(P_3host3[,2:11])

P_3host <- ggord(ord, P_3host3$Condition3,cols = c("green",'blue','red'),obs.scale = 0, var.scale = 1,arrow=0.2,veclsz =0.2,vec_ext =8,size=2.5,scannf=TRUE,nfposi=3,txt=3, ext=1.1, nfac = 1,grp_title = "Condition")+theme_minimal()+theme(panel.grid = element_blank(),axis.text = element_blank(),axis.title = element_blank(),plot.title = element_blank(),legend.title = element_text(hjust =0.1,size=10),legend.text = element_text(size=9),legend.position="top",plot.margin = unit(c(0,3,4,5),"cm"))+ geom_ord_ellipse(ellipse_pro = .95, lty=5)

my_comparisons <- list(c("HC", "Stable stage"), c("Stable stage", "Active stage"),c("HC", "Active stage"))

compare_means(Scores~Condition3,data= P_3host1_scores[c(-21:-40),],comparisons= P_3host1_comparisons)

P_3host1_x <- ggviolin(P_3host1_scores[c(-21:-40),], x="Condition3", y="Scores",fill= "Condition3",palette = c("green","blue", "red"), trim =TRUE,position = position_dodge(0.3), alpha =0.3, add = c("boxplot"), ylab = "Separation of SLE",add.params=list(color='black', size=0.2))+theme_bw()+rremove("legend")+rremove("grid")+rremove("y.axis")+ rremove("y.ticks")+rremove("y.text")+rremove("ylab")+ theme(panel.border= element_blank(),axis.line = element_line(size=0.5, colour = "black"))+font("xlab",size = 12,color = "black")+font("x.text",size = 12,color = "black")+ stat_compare_means(Scores~Condition3,data= P_3host1_scores[c(-21:-40),],label = "p.signif",vjust=5,comparisons= P_3host1_comparisons,size=5)+rotate()

compare_means(Scores~Condition3, data= P_3host1_scores[c(-1:-20),], comparisons= P_3host1_comparisons)

P_3host1_y <-ggviolin(P_3host1_scores[c(-1:-20),], x="Condition3", y="Scores", fill= "Condition3",palette = c("green","blue", "red"), position = position_dodge(0.3),alpha =0.3,trim =TRUE,add = c("boxplot"),ylab = "Separation of SLE",add.params =list(color= 'black',size=0.3))+ theme_bw()+rremove("legend")+rremove("grid")+ rremove("x.axis")+ rremove("x.ticks")+rremove("x.text")+rremove("xlab")+theme(panel.border = element_blank(),axis.line = element_line(size=0.5, colour = "black"))+font("ylab", size = 12,color = "black")+font("y.text",size = 12,color = "black")+stat_compare_means(label = "p.signif",vjust=1,hjust=-1,label.y = c(2.0, 2.3, 2.6),comparisons= P_3host1_comparisons, size = 5)

P_3host1_x_grob <- ggplotGrob(P_3host1_x)

P_3host1_y_grob <- ggplotGrob(P_3host1_y)

P_3host +annotation_custom(grob = P_3host1_x_grob, xmin =-6, xmax =10.5, ymin = -11, ymax = -5)+annotation_custom(grob = P_3host1_y_grob, xmin =-12, xmax =-6, ymin = -3, ymax = 7)

P_2host1 <-read.csv("10_host.csv")

P_2host1_scores <- read.csv("host1_PCA_data_output.csv")

P_2host3<- P_2host1[,c(-1,-2)]

rownames(P_2host1)= P_2host1[,1]

P_2host2<- P_2host1[,c(-1,-2,-3)]

ord <- PCA(P_2host3[,2:11])

P_2host <-ggord(ord, P_2host3$Condition2,cols = c('blue','red'),obs.scale = 0, var.scale = 1,arrow=0.2,veclsz =0.2,vec_ext =7,size=2.5,scannf=TRUE,nfposi=3,txt=3, ext=1.1,nfac = 1,grp_title = "Condition")+theme_minimal()+theme(panel.grid = element_blank(),axis.text = element_blank(),axis.title = element_blank(),plot.title = element_blank(),legend.title = element_text(hjust =0.1,size=10),legend.text = element_text(size=9),legend.position="top", plot.margin = unit(c(0.5,1,4,5),"cm"))+geom_ord_ellipse(ellipse_pro = .95, lty=5)

compare_means(Scores~Condition2,data= P_2host1_scores[c(-21:-40),])

P_2host1_x <- ggviolin(P_2host1_scores[c(-21:-40),], x="Condition2", y="Scores", fill="Condition2",palette = c("blue", "red"), trim =TRUE,position = position_dodge(0.3), alpha =0.3, add = c("boxplot"), ylab = "Separation of SLE",add.params=list(color='black', size=0.2))+theme_bw()+rremove("legend")+rremove("grid")+rremove("y.axis")+ rremove("y.ticks")+rremove("y.text")+rremove("ylab")+theme(panel.border = element_blank(),axis.line = element_line(size=0.5, colour = "black"))+font("xlab",size = 12,color = "black")+font("x.text",size = 12,color = "black")+stat_compare_means(label = "p.signif",vjust=-1.2,size=5,hjust=1)+rotate()

compare_means(Scores~Condition2, data= P_2host1_scores[c(-1:-20),])

P_2host1_y <-ggviolin(P_2host1_scores[c(-1:-20),], x="Condition2", y="Scores", fill= "Condition2",palette = c("blue", "red"), position = position_dodge(0.3),alpha =0.3,trim =TRUE,add = c("boxplot"),ylab = "Separation of SLE",add.params=list(color='black', size=0.3))+theme_bw()+rremove("legend")+rremove("grid")+rremove("x.axis")+rremove("x.ticks")+rremove("x.text")+rremove("xlab")+theme(panel.border = element_blank(),axis.line = element_line(size=0.5, colour = "black"))+font("ylab", size = 12,color = "black")+ font("y.text",size = 12,color = "black")+stat_compare_means(label = "p.signif",vjust=2, hjust=-0.5,size = 5)

P_2host1_x_grob <- ggplotGrob(P_2host1_x)

P_2host1_y_grob <- ggplotGrob(P_2host1_y)

P_2host + annotation_custom(grob = P_2host1_x_grob, xmin =-6, xmax =7, ymin = -9, ymax = -4.2)+annotation_custom(grob = P_2host1_y_grob, xmin =-11, xmax =-6.2, ymin = -3, ymax = 5.5)

###Correlation analysis of differential expressed genes with core viruses and the graph###

Gene_species =read.csv("Gene_species.csv", header=TRUE)

rownames(Gene_species)<- Gene_species[,1]

Gene_species <- Gene_species[,-1]

#Computes a matrix of Spearman’s rho rank correlation coefficients for all possible pairs of columns of a matrix.

Gene_species1<-rcorr(t(Gene_species),type ="spearman")

Gene_species_correlation(spearman) <- Gene_species1$r #Get Spearman’s correlation coefficients

write.csv(Gene_species_correlation(spearman),"gene_species_correlation(spearman)_r.csv")

Gene_species_correlation(spearman)_p <- Gene_species1$P #Get P values

write.csv(Gene_species_correlation(spearman)_p,"gene_species_correlation(spearman)_p.csv")

gene_species_correlation_p =read.csv("gene_species_correlation(spearman)_p1.csv", header=TRUE)

rownames(gene_species_correlation_p)<- gene_species_correlation_p[,1]

gene_species_correlation_p <- gene_species_correlation_p[,-1]

gene_species_correlation_p[gene_species_correlation_p > 0.05]=0

write.csv(gene_species_correlation_p,"gene_species_correlation(spearman)_p1_0.05.csv")

gene_species_correlation_r =read.csv("gene_species_correlation(spearman)_r1.csv", header=TRUE)

rownames(gene_species_correlation_r)<- gene_species_correlation_r[,1]

gene_species_correlation_r <- gene_species_correlation_r[,-1]

gene_species_correlation_r[abs(gene_species_correlation_r) < 0.6]=0

write.csv(gene_species_correlation_r,"gene_species_correlation(spearman)_r1_0.6.csv")

gene_species_result =read.csv("gene_species_0.6_0.05.csv", header=TRUE)

rownames(gene_species_result)<- gene_species_result[,1]

gene_species_result <- gene_species_result[,-1]

pheatmap(gene_species_result,clustering_distance_rows="correlation",clustering_distance_cols="correlation",color = colorRampPalette(c("blue", "white", "red"))(50), show_colnames = F,fontsize_row=14,fontsize_col =4) #Graph of correlation analysis

#Get the data of above graph.

gene_species_result =read.csv("gene_species_0.6_0.05.csv", header=TRUE)

rownames(gene_species_result)<- gene_species_result [,1]

gene_species_result <- gene_species_result [,-1]

gene_species_result_hc=hclust(dist(gene_species_result),method ="correlation")

gene_species_result_row_order= gene_species_result_hc$order

gene_species_result1= gene_species_result[gene_species_result_row_order,]

gene_species_result1=melt(gene_species_result1)

write.csv(gene_species_result1,"1.csv")
